# Comparative Proteomics and Phosphoproteomics Analysis Reveal the Possible Breed Difference in Yorkshire and Duroc Boar Spermatozoa

**DOI:** 10.3389/fcell.2021.652809

**Published:** 2021-07-16

**Authors:** Yongjie Xu, Qiu Han, Chaofeng Ma, Yaling Wang, Pengpeng Zhang, Cencen Li, Xiaofang Cheng, Haixia Xu

**Affiliations:** ^1^College of Life Science, Xinyang Normal University, Xinyang, China; ^2^Institute for Conservation and Utilization of Agro-Bioresources in Dabie Mountains, Xinyang Normal University, Xinyang, China; ^3^Xinyang Animal Disease Control and Prevention Center, Xinyang, China

**Keywords:** spermatozoa, breed, sperm motility, proteomics, phosphoproteomics, iTRAQ labeling

## Abstract

Sperm cells are of unique elongated structure and function, the development of which is tightly regulated by the existing proteins and the posttranslational modifications (PTM) of these proteins. Based on the phylogenetic relationships of various swine breeds, Yorkshire boar is believed to be distinctly different from Duroc boar. The comprehensive differential proteomics and phosphoproteomics profilings were performed on spermatozoa from both Yorkshire and Duroc boars. By both peptide and PTM peptide quantification followed by statistical analyses, 167 differentially expressed proteins were identified from 1,745 proteins, and 283 differentially expressed phosphopeptides corresponding to 102 unique differentially phosphorylated proteins were measured from 1,140 identified phosphopeptides derived from 363 phosphorylated proteins. The representative results were validated by Western blots. Pathway enrichment analyses revealed that majority of differential expression proteins and differential phosphorylation proteins were primarily concerned with spermatogenesis, male gamete generation, sperm motility, energy metabolism, cilium morphogenesis, axonemal dynein complex assembly, sperm–egg recognition, and capacitation. Remarkably, axonemal dynein complex assembly related proteins, such as SMCP, SUN5, ODF1, AKAP3, and AKAP4 that play a key regulatory role in the sperm physiological functions, were significantly higher in Duroc spermatozoa than that of Yorkshire. Furthermore, phosphorylation of sperm-specific proteins, such as CABYR, ROPN1, CALM1, PRKAR2A, and PRKAR1A, participates in regulation of the boar sperm motility mainly through the cAMP/PKA signal pathway in different breeds, demonstrating that protein phosphorylation may be an important mechanism underlying the sperm diversity. Protein–protein interaction analysis revealed that the 14 overlapped proteins between differential expression proteins and differential phosphorylation proteins potentially played a key role in sperm development and motility of the flagellum, including the proteins ODF1, SMCP, AKAP4, FSIP2, and SUN5. Taken together, these physiologically and functionally differentially expressed proteins (DEPs) and differentially expressed phosphorylated proteins (DPPs) may constitute the proteomic backgrounds between the two different boar breeds. The validation will be performed to delineate the roles of these PTM proteins as modulators of Yorkshire and Duroc boar spermatozoa.

## Introduction

In the modern swine production, artificial insemination (AI) is one of the most critical technologies in the genetic improvement of porcine herds. Sperm quality, as an important boar reproductive trait with moderate to low heritability, is crucial for insuring the success of AI, which is influenced by both environmental and genetic factors (Marques et al., 2017; Gao et al., 2019). Duroc, Yorkshire, and Landrace are the most frequently used pigs in commercial production and have favorable growth performance. Due to the various origins, these breeds not only are significantly different in the aspect of meat productive traits but also have great differences in reproductive traits, such as male fertility traits, including sperm number, sperm-fertilizing capacity, sperm motility, concentration, and vitality, and semen volume (Koh et al., 1976; Kasimanickam and Kastelic, 2016; Shanmugam et al., 2016). For instance, Large White and Landrace boars had higher total sperm number and ejaculate volume, but less sperm motility and concentration than Duroc breeds (Ciereszko et al., 2000; Smital, 2009). Although it is known that there are differences in semen traits among the different porcine breeds, the diversity in the molecular mechanism of the genetic background has not been well characterized. Thereby, providing the impetus to understanding the genetic background associated with sperm quality traits in different breeds is of great benefit to improve the genetic selection for these traits and accelerate genetic progress.

Mature mammalian sperm are highly differentiated haploid cells which almost silenced at the level of transcriptional and translational regulation. As the carrier of patrilineal genes, sperm cells are unique in elongated structure and function and are tightly regulated by the existing proteins and the posttranslational modifications (PTM), which have served as a resource pool for screening of key targets involved in sperm motility regulation (Samanta et al., 2016; Dai et al., 2019b; Gadadhar et al., 2021). Owing to this essential role in sperm cells, the study of sperm protein is of great significance for clarifying the physiological process of mammalian spermatogenesis and conception. Due to the extensive application of “omics” technology for system biology, tremendous progress has been made in recent years on protein function research through global proteome analysis and its complex interaction in response to specific disturbance (Larance and Lamond, 2015; Dai et al., 2019a). Proteomics has also been widely used in the study of human and other animal sperm, which can reflect the protein composition, distribution, and function of sperm cells from a macro perspective, and indicated that sperm proteins play essential functional roles and contribute to vital biological processes such as spermatogenesis, sperm motility, sperm capacitation, fertilization, and male infertility (Chalmel and Rolland, 2015; Baker, 2016; Maciel et al., 2019; Agarwal et al., 2020; Martin-Hidalgo et al., 2020). Therefore, quantitative proteomics analysis is a potent tool for understanding the quality variation in sperm functions.

On a genome analysis of cumulative nucleotide differences, Yorkshire pigs differed significantly from Duroc, consistent with being the two breeds with a rather distant relationship based on phylogenetic analyses (Kim et al., 2015). The semen trait differences in sperm motility and capacitation, fertility, and semen volume among the porcine breeds were studied in the previous experiments (Ciereszko et al., 2000; Smital et al., 2004; Flowers, 2008; Smital, 2009). The protein function of sperm-fertilizing and its relationship with different boar breeds have been increasingly a focus in these years, and the identification of differentially expressed proteins in spermatozoa can help to clarify the molecular mechanisms of the genetic background from different boar breeds. Xinhong et al. (2018) firstly showed an iTRAQ-based proteomics analysis of sperm proteins in Meishan and Duroc boar species and provided significant information for elucidating the molecular basis responsible for variety-specific differences in pig reproductive efficiency. Despite that protein regulation at the phosphorylation level plays crucial functions in sperm, very few studies have reported the diversities in phosphoproteins associated with sperm motility between different porcine breeds. The precise genetic mechanisms behind the sperm vitality and motility in different breeds remain unclear, and there was no comprehensive analysis of the proteome or phosphoproteome determining breed differences in boar sperm. In this research, we used a global analysis of iTRAQ-based quantitative proteome coupled with phosphopeptide-enrichment strategies to reveal the boar spermatozoa proteome and find the PTMs associated with the breed differences using Yorkshire and Duroc boar spermatozoa. Our results show that the porcine sperm protein phosphorylation status relates exclusively to sperm structure and motility in different breeds, thus supporting its importance for phosphorylation status changes in distinguishing breed specificity. Moreover, this research will provide a new insight to understanding the molecular basis of the differences in pig reproductive efficiency between Duroc and Yorkshire breeds.

## Materials and Methods

### Semen Sources and Preparation

Fresh boar semen was obtained from Hongzhan Pig Breeding Farm (Xinyang, Henan, China). All boars were fed similar amounts of a common ration, with management and nutrition in accordance with good industry practices. Semen was collected concurrently from Duroc and Yorkshire boars (four boars per breed), with all boars 18–24 months old at the time of semen collection. Semen was treated according to the method as described by Kasimanickam and Kastelic (2016). Briefly, initial post-collection motility was consistently ≥ 80%, the minimum content of sperm with normal morphology was 80%, and the sperm density was 2.0∼3.5 × 10^8^/ml. The sperm-rich fraction was diluted in Beltsville thawing solution (BTS) buffer (1:1 volume) and transported to the laboratory at 37°C within 1 h. Upon arrival, diluted semen was placed in 50-ml Falcon tubes and centrifuged at 1,000 *g* for 20 min (4°C) to separate seminal plasma. Sperm were washed twice using BTS at 1,000 *g* for 20 min (4°C). The sperm pellet was resuspended in BTS, then aliquoted into microcentrifuge tubes, and centrifuged at 16,000 *g* for 10 min (4°C). The supernatant was completely discarded, and the sperm precipitate was fast frozen in liquid nitrogen and stored in the refrigerator at –80°C until used. All procedures were carried out in accordance with the Animal Ethical Treatment Guidelines and were approved by the Animal Care Commission of the College of Life Science, Xinyang Normal University, China.

### Sperm Protein Extraction

Immediately after collection, 15-ml tubes were fully filled from each ejaculation/segment and were centrifuged twice at 1,500 *g* for 10 min. Sperm pellets were lysed in STD buffer (1 mM DTT, 4% SDS, 150 mM Tris–HCl pH 8.0) containing a 1% protease inhibitor cocktail (Roche). The sample was homogenized on ice by sonication with an ultrasonic cell crusher (DH92-IIN, Toshiba) 10 times (10-s pulse on/15-s pulse off). Samples were centrifuged at 16,000 *g* for 45 min at 4°C, and the supernatants were collected. The protein concentration was quantified by the BCA assay (Beyotime, P0012S, Shanghai, China) with BSA protein as standard.

### Protein Digestion, iTRAQ Labeling, and Peptide Fractionation

Protein digestion was performed according to the previously filter-aided sample preparation (FASP) procedure (Wisniewski et al., 2009). Briefly, 200 μg of each protein sample was mixed with 200 μl UA buffer (8 M urea, 150 mM Tris–HCl pH 8.0) and concentrated for 15 min at 14,000 *g* using 10-kDa ultrafiltration centrifuge tubes. The retentates were resuspended in 200 μl UA buffer and concentrated for another 15 min at 14,000 *g* at room temperature. Then, 100 μl of 0.05 M IAA in UA buffer was added to inhibit reduced cysteine residues, and the samples were incubated for 30 min in darkness and concentrated for 10 min at 14,000 *g*. Subsequently, the filters were washed with 100 μl of UA buffer twice and spun for 10 min at 14,000 g, and then washed twice with 100 μl of DS buffer (50 mM trimethylammonium bicarbonate at pH 8.5) and spun for 10 min at 14,000 *g*. Finally, the protein suspensions were digested with 40 μl 50 ng/μl sequencing-grade trypsin (Roche, IN, United States) (2 μg trypsin in 40 μl DS buffer) overnight at 37°C, and the resulting peptides were collected as a filtrate. The peptide content was estimated by UV light spectral density at 280 nm (Wisniewski et al., 2009).

**FIGURE 1:**
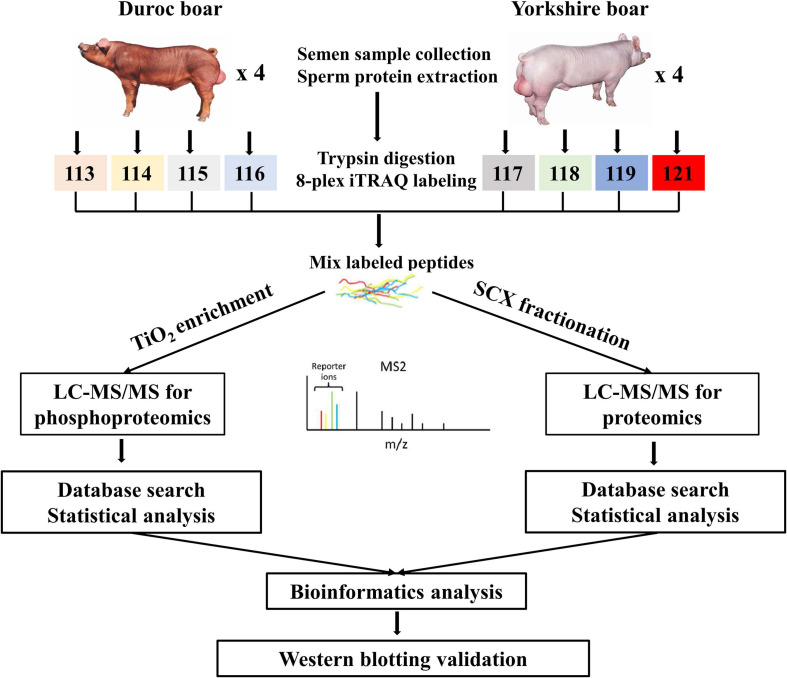
Experimental design and schematic diagram of the workflow. Boar sperm was chosen for analysis of the differential proteomes between the Duroc and Yorkshire pig breeds. A total of eight boars were analyzed by iTRAQ 8Plex-based parallel quantitative proteomics and phosphoproteomics, using the LC-MS/MS workflow. After thorough statistical analyses of the proteomics data, the differentially expressed proteins and phosphoproteins/sites were conducted for the subsequent bioinformatics analysis and some of the important differentially expressed proteins were selected for validation of the discovery results by western blotting.

For proteome analysis, the resulting peptide mixture was labeled using the iTRAQ Reagent-8Plex Multiplex kit (AB SCIEX, Foster City, CA, United States) according to the manufacturer’s instructions. In detail, each iTRAQ reagent was dissolved in 70 μl of ethanol and added to different peptide mixtures. The 80-μg digested peptides from each sample were incubated with specific iTRAQ reagents (iTRAQ reagents 113, 114, 115, and 116 used for four Duroc sperm samples, and iTRAQ reagents 117, 118, 119, and 121 for four Yorkshire sperm samples, respectively) for 1 h at room temperature. The digested peptides used for proteomic and phosphoproteomic analysis were labeled separately. After labeling, samples were multiplexed and concentrated in a vacuum concentrator for further identification and quantification by LC-MS/MS. Proteins showing different abundances between the Yorkshire and Duroc groups, as shown in Figure 1, were subjected to bioinformatics analysis, and quantification was validated by Western blot.

Additionally, to identify more proteins for proteome analysis, Strong cation exchange (SCX) was applied to separate the mixed peptides using AKTA Purifier 100 (GE Healthcare, Fairfield, CT, United States) as previously described (Dong et al., 2017). In brief, the vacuum-dried iTRAQ-labeled peptide mixture was reconstituted in 2 ml buffer A (25% CAN and 10 mM KH_2_PO_4_ pH 3.0), loaded onto a Polysulfoethyl 4.6 mm × 100 mm column (5 μm, 200 Å) (PolyLC Inc., Columbia, MD, United States), and eluted at 0.7 ml/min with the following concentrations of buffer B (25% CAN, 500 mM KCl, and 10 mM KH_2_PO_4_ pH 3.0) successively: 0% buffer B from 0 to 25 min; 10% buffer B for 32 min; 20% buffer B for 42 min; 45% buffer B for 47 min; 100% to 0% buffer B from 52 to 60 min; and 0% buffer B for 75 min. After separation, the 33 fractions collected were combined to 10 fractions according to SCX chromatogram, then desalted on standard density Empore SPE C_18_ cartridges (Sigma, St. Louis, MO, United States) with inner diameter 7 mm and volume 3 ml, concentrated by vacuum centrifugation.

### Phosphopeptide Enrichment

Phosphopeptide enrichment was done as described previously (Du et al., 2017). Briefly, the vacuum-dried peptide mixture was resuspended in 500 μl loading buffer (2% glutamic acid/2% TFA/65% ACN). TiO_2_ beads were added, and the samples were agitated by end-over-end rotation at 20°C for 40 min. The phosphopeptide-bound beads were collected by brief centrifugation, washed three times with 50 μl of buffer I (3% TFA/30% ACN), and then washed three times with 50 μl of buffer II (0.3% TFA/80% ACN) to remove any non-adsorbed material. Phosphopeptides were finally eluted with 50 μl of elution buffer (15% NH_4_OH/40% ACN), dried, and stored at –80°C until further analysis.

### LC-MS/MS Analysis Based on Q Exactive Mass Spectrometry

LC-MS/MS analyses were performed on a Q Exactive mass spectrometer coupled to an EASY-nLC 1000 nano-HPLC system (Proxeon Biosystems, Thermo Fisher Scientific, Waltham, MA, United States) as described previously (Wei et al., 2018). Briefly, each dried peptide fraction or dried TiO_2_-enriched phosphopeptides were reconstituted in 20 μl of 0.1% formic acid and loaded on a Thermo Scientific EASY column (2 cm × 100 μm, 5 μm C18 resin) equilibrated with 95% Buffer A (0.1% formic acid), and then the peptides were loaded and separated on a C18 column (25 cm × 75 μm, 3 μm C18 resin) at a flow rate of 250 nl/min. Peptide and phosphopeptide mixtures were eluted using buffer A (0.1% formic acid) and buffer B (100% acetonitrile and 0.1% formic acid) under a 170-min gradient with a flow rate of 250 nl/min (0–30% buffer B for 120 min, 30–50% buffer B for 25 min, 50–100% buffer B for 15 min, and finally 100% buffer B for 10 min).

For MS data acquisition, the eluted peptides and phosphopeptides were analyzed in positive ion mode on a Q Exactive mass spectrometer (Thermo Finnigan, San Jose, CA, United States) using data-dependent acquisition. The full mass scan was acquired by the Orbitrap mass analyzer from m/z 300 to 1800 with a resolution of 70,000 at m/z 200, and the AGC target was set to 3 × 10^6^ with a max injection time of 20 ms. The 10 most intense parent ions were fragmented by higher-energy collisional dissociation (HCD). The MS/MS scans were also acquired by the Orbitrap with 17,500 resolution at m/z 200, and the AGC target was set to 2 × 10^4^ with a max injection time of 60 ms. System control and data collection were performed by Xcalibur software (Thermo Fisher Scientific, United States). The mass spectrometry raw data have been deposited to the ProteomeXchange Consortium^1^ *via* the iProX partner repository with the dataset identifier PXD025607 (Ma et al., 2019). The raw MS data files contain 15 files. P230-01.msf and P230-PH-3.msf are the search engine output files for proteome and phosphoproteome analysis, respectively. P230-1.raw, P230-2.raw, P230-3.raw, P230-4.raw, P230-5.raw, P230-6.raw, P230-7.raw, P230-8.raw, and P230-9.raw are the mass spectrometer output files of the proteome. P230-PH-1.raw, P230-PH-2.raw, and P230-PH-3.raw are the mass spectrometer output files of the phosphoproteome.

### Mass Spectrometry Data Analysis

The raw MS/MS files were processed with Proteome Discoverer version 1.4 (Thermo Fisher Scientific, United States) and subjected to an in-house UniProt Sus scrofa (pig) protein database (UniProt 2018_01_27; 50,008 sequences; updated on 1-27-2018) searching using Mascot Server Version 2.2 (Matrix Science, London, United Kingdom). For proteome analysis, the parameters for database searching were set as follows: enzyme, trypsin; iTRAQ 8Plex labels, the N-terminal and lysine residues; maximum missed cleavages, 2; fixed modification, carbamidomethylation of cysteine residues; peptide mass tolerance, ±20 ppm; MS/MS tolerance, 0.1 Da; variable modifications, oxidation of methionine. For the phosphoproteome, parameters for protein identification were set as follows: enzyme, trypsin; mass values, monoisotopic; peptide mass tolerance, ±20 ppm; MS/MS tolerance, 0.1 Da; maximum missed cleavage, 2; iTRAQ 8Plex labels, the N-terminal and lysine residues; fixed modification, carbamidomethylation of cysteine residues; variable modifications, oxidation of methionine and phosphorylation of threonine/serine/tyrosine; instrument type, ESI-TRAP. The decoy database pattern was set as the reverse of the target database. Trypsin with full enzyme specificity and only peptides with a minimum length of six amino acids were selected. Protein identification was succeeded by at least one unique peptide identification. The minimum MaxQuant score of phosphorylation sites was 40. All reported data were based on 99% confidence for protein, peptide, and phosphorylation site identifications as determined by an FDR of ≤0.01 (Sandberg et al., 2012). Protein identification was supported by at least one unique peptide identification. PhosphoRS score >50 and PhosphoRS site probability >75% indicate that a site is truly phosphorylated (Olsen et al., 2006).

Peptides with different amino acid sequences or modifications were identified as unique peptides. For quantification, only unique peptides were considered, and it was performed simultaneously with protein identification using Proteome Discoverer software. The log_2_ values of the measured precursor intensities were normalized by the median values across an entire labeling experiment to correct for protein abundance variation (Unwin et al., 2010). A two-sample *t-*test was carried out within SPSS 18.0. Proteins or phosphopeptides with *p* < 0.05 after Benjamini and Hochberg adjustment and fold-change ratios ≥1.3 or ≤0.77 were considered as differentially expressed proteins (DEPs) or differentially expressed phosphopeptides (DEPPs).

### Bioinformatics Analysis

The biological functions of identified DEPs and differential phosphorylation proteins (DPPs) were annotated using GO enrichment^2^ and the KEGG pathway^3^. To understand these DEPs and DPPs in terms of the published literature, interactions among them in relation to function and biological pathways were determined using the IPA tool. Interactions among all the related DEPs and DPPs were constructed using the program STRING^4^. The STRING program was set to show no more than 10 interactions and medium confidence.

### Western Blotting

Sperm lysates were run on SDS-PAGE gels, and immunoblotting was performed according to our previous procedure (Xu et al., 2012). The primary antibodies used were as follows: mouse anti-HNRNPK (ab39975, Abcam, Cambridge, MA, United States), rabbit anti-AKAP3 (13907-1-AP, Proteintech, Wuhan, China), rabbit anti-ODF1 (24736-1-AP, Proteintech), rabbit anti-PTGDS (10754-2-AP, Proteintech), rabbit anti-AKAP4 (24986-1-AP, Proteintech), rabbit anti-GK (13360-1-AP, Proteintech), and mouse anti-GAPDH (AF5009, Beyotime). Immunoblots were repeated at least three times for each sample from four Duroc and Yorkshire pigs, respectively. Detection of proteins was performed using enhanced chemiluminescence (ECL). Finally, the blot was imaged using a FluorChem M multicolor fluorescence Western blot imaging system (ProteinSimple, San Jose, CA, United States).

## Results

### Proteomic and Phosphoproteomic Profiles of Porcine Spermatozoa

**FIGURE 2:**
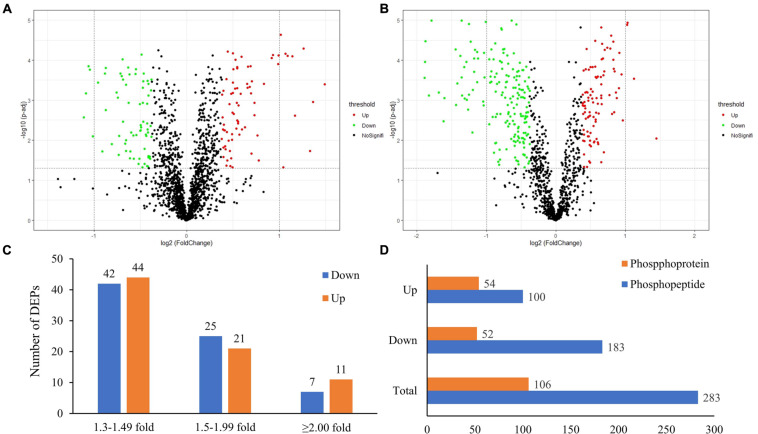
The global proteome and phosphoproteome analysis of Yorkshire and Duroc boar spermatozoa. **(A)** Volcano plot of the quantified proteins in all biological replicates (the significant downregulated and upregulated protein shown in the green and red plots, respectively). **(B)** Volcano plot of the quantified phosphorylated sites in all biological replicates after TiO_2_ enrichment (the significant downregulated and upregulated phosphorylated sites shown in the green and red plots, respectively). **(C)** The fold-change distribution of DEPs. **(D)** Summary of the DEPPs and DPPs between Yorkshire and Duroc boar spermatozoa.

**FIGURE 3:**
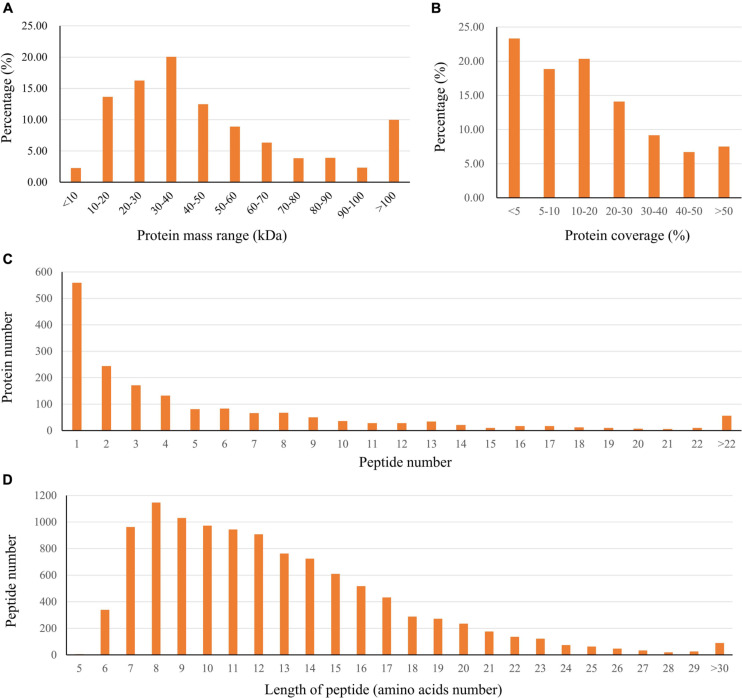
Characteristics of the identified unique peptides in boar sperm samples. **(A)** The protein mass distribution. **(B)** The protein coverage. **(C)** The distribution of unique peptide number. **(D)** The distribution of peptides based on their length.

In this study, the global protein expression and phosphorylation events were compared between Duroc and Yorkshire boar spermatozoa using 8Plex iTRAQ-based quantitative proteomics. Four independent biological replicates were performed for each sperm protein sample for iTRAQ labeling. We identified 10,876 unique peptides from 187,889 spectra corresponding to 1,745 protein species (Supplementary Data 1, 2, <1% FDR). The 1,745 identified proteins were annotated with UniProtKB (ENSEMBL) databases, corresponding to 1,697 (97.2%) full protein and 48 (2.8%) protein fragment annotations. However, 384 identified proteins were cataloged as uncharacterized protein isoforms due to the scarcity of porcine protein databases. Among these proteins, 1,738 were successfully quantified, of which 150 proteins were detected as DEPs between the two groups as a cutoff of 1.3-fold change (Figure 2A). The predicted molecular weights (MW) of the identified proteins vary widely with a range from 1.7 to 763.6 kDa with a mean of 57.3 kDa (Figure 3A). The sequence coverage of peptides (Figure 3B) and the distribution of the peptide number (Figure 3C) and peptide length (Figure 3D) were also provided. More than 67.9% of the identified peptides were detected from at least two unique peptides. In addition, the protein sequence coverage with >50, 40–50%, 30–40%, 20–30%, 10–20%, 5–10%, and under 5% variation accounted for 7.51, 6.70, 9.17, 14.10, 20.34 18.85, and 23.32%, respectively, of the total identified proteins (Figure 3B).

**FIGURE 4:**
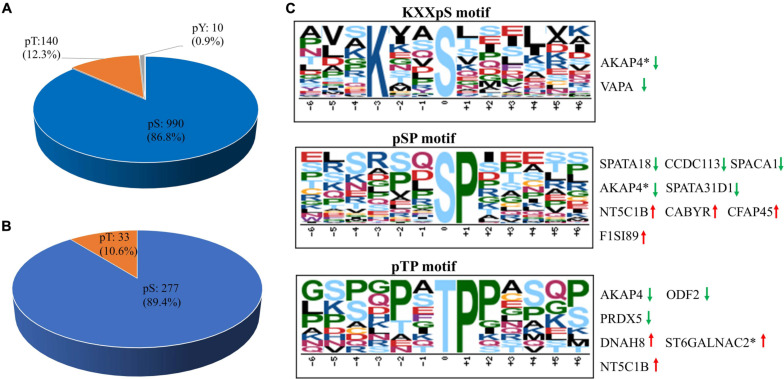
The distribution and phosphorylation motif enrichment of the identified phosphorylation events. **(A)** The distribution of the phosphorylated serine (pS), threonine (pT), and tyrosine (pY) among the identified phosphorylation events. **(B)** The distribution of pS and pT sites of the differentially expressed phosphopeptides in boar sperm. **(C)** The Significantly enriched phosphorylation motif from all phosphorylation events. The height and color of the residues represent the frequency occurring at the respective positions and their physicochemical properties, respectively. Red arrow represents upregulated phosphosites, green arrow represents downregulated phosphosites, and asterisk represents that two or more phosphosites own the same the phosphorylation motif in the same protein.

Using PhosphoRS probability cutoff >75%, 1,140 phosphosites were identified from 1,064 unique phosphopeptides mapping to 363 proteins of porcine sperm (Supplementary Data 3), of which 283 unique phosphopeptides were detected to be differentially expressed between the two breeds as a cutoff of 1.3-fold change (Figure 2B). Amid these phosphopeptides, 705 peptides only showed one phosphorylation site, 274 peptides showed two, and 49 peptides showed three or more. On analysis of phosphosites of the phosphorylated proteins, 208 proteins were identified to be phosphorylated at a single site on the protein sequence, and 155 proteins were phosphorylated at two or more. AKAP4, as the most prominent example, is identified as being targeted for 45 phosphorylated sites including 55 phosphopeptides (Supplementary Figure 1). As would be expected, consistent with previous published reports, the 1,140 assigned sites included 990 phosphorylated serine (pS), 140 phosphorylated threonine (pT), and 10 phosphorylated tyrosine (pY) residues (ratios of 86.8, 12.3, and 0.9%), respectively, indicated in Figure 4A. These results can tremendously contribute to the porcine sperm protein phosphorylation database for future study.

**FIGURE 5:**
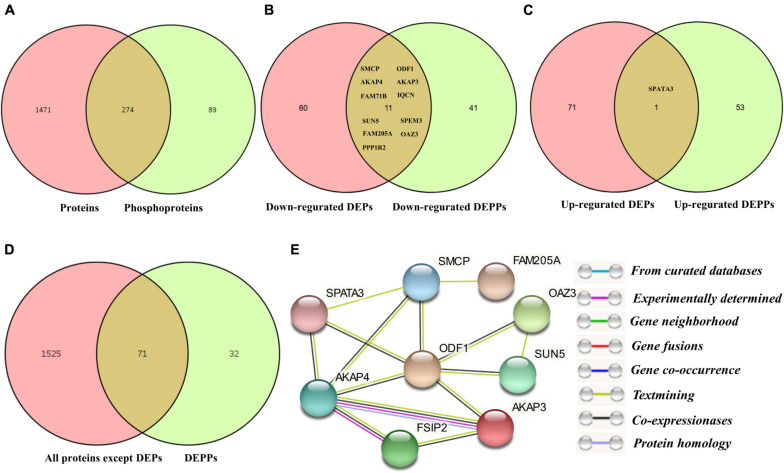
The correlation analysis between DEPs and DEPPs. **(A)** Venn diagram of the correlation numbers between quantified proteins and phosphoproteins. **(B)** Venn diagram of the correlation numbers between downregulated DEPs and downregulated DEPPs. **(C)** Venn diagram of the correlation numbers between upregulated DEPs and upregulated DEPPs. **(D)** Venn diagram of the intersection numbers between total identified porcine boar sperm proteins and DEPPs. **(E)** The protein–protein interaction network of the 14 overlapped proteins between DEPs and DEPPs.

Comparing the number of identified proteins and phosphorylated proteins, all the proteins identified in proteomics (1,745) and phosphoproteomics (363) sets were demonstrated in the Venn diagram (Figure 5A). Only 274 proteins (75.5%) were overlapped between non-modified proteins and phosphoproteins, while ∼24.5% of the total phosphoproteins were not detected in the global proteomics analysis. The explanation for this result was that most of the identified phosphorylated proteins are expressed in relatively low abundance in porcine sperm and identified mainly depending on the specificity of the enrichment strategy for phosphopeptides. In unenriched fractions, there are more redundant peptides mainly derived from a very small number of highly abundant proteins, such as sperm structure proteins (e.g., PRM-1, several tubulin family members, FN1, and ODF1) and sperm–egg interaction proteins (e.g., SPACA1, SPESP1, PSP-I/II, and AQN-3), which decrease the probability of low-abundance-protein identification. This comparison also demonstrated that the extensive biochemical heterogeneity of sperm proteins led to the technical challenge and complexity of sperm proteome for its high dynamicity and diversity.

To obtain a global view of the biological function of the identified proteins and phosphoproteins, enriched GO terms were performed using the Panther classification system^5^. The cutoff of the *p*-value is set to 0.05, and terms of the same category are ordered by *p*-values. Analysis indicated that this global porcine sperm proteome returned dominant terms of cytoplasm, membrane, mitochondrion, cell periphery, and cell projection among the top GO cellular compartment categories when ranked based on number of annotated proteins (Supplementary Figure 2A). The notable enrichment GO biological processes were metabolic process, biological regulation, regulation of cellular process, stimulus response, regulation of metabolic process, cellular component assembly, and reproduction (Supplementary Figure 2B). Additional categories of direct relevance to sperm physiology/function included reproductive process, cell communication, and spermatogenesis. The dominant GO molecular functions represented in the porcine sperm proteome included that of catalytic activity, hydrolase activity, small molecule binding, nucleotide binding, and oxidoreductase activity, with some 313, 164, 159, 143, and 72 proteins mapping to each of these respective categories (Supplementary Figure 2C). Phosphoproteins were overrepresented from cell organelles, such as integral component of membrane, intrinsic component of membrane, cytoskeletal part, cell projection, and microtubule cytoskeleton, and more involved in biological regulation, stimulus response, reproductive process, regulation of biological process, and spermatogenesis, and with the function of catalytic activity, hydrolase activity, small molecule binding, and anion binding.

To gain insight into the potential categorization of mycobacterial kinase substrates, the Motif-X algorithm was used to analyze the phosphorylation motifs with a relative occurrence rate threshold of 3% and a possibility threshold of *p* < 10^–6^ (Chou and Schwartz, 2011). Due to lack of a specialized annotated kinase/phosphatase motif database in pigs, the generated motifs were matched against the Human Protein Reference Database^6^. We identified predominantly three phospho-motifs, KXXpS for PKCs and AKT (Ren et al., 2018), pSP/pTP for GSK-3, CDK, and MAPK families (Figure 4C). Thirty-seven substrates shared the KXXpS motif, of which three phosphopeptides (two proteins, AKAP4 and VAPA) were lowly phosphorylated in Yorkshire boar spermatozoa. In this category, the two AKAP4 phosphopeptides (AVSKIASEMAHDA and AAEKGYSVGDLLQ) involved in sperm motility and regulation of signal transduction pathways were found to be 1.33-fold and 3.03-fold, respectively, more highly phosphorylated in Duroc boar spermatozoa. Furthermore, 36 substrates shared the pSP motif, of which five phosphopeptides (four proteins, SPATA18, SPACA1, AKAP4, and SPATA31D1) were lowly phosphorylated in Yorkshire boar spermatozoa and four proteins (NT5C1B, CABYR-1, CFAP45, and TMEM202) were lowly phosphorylated in Duroc boar spermatozoa. Interestingly, 11 kinase substrates shared pTP, of which four (three proteins, DNAH8, St6galnac2, and NT5C1B) were highly phosphorylated in Yorkshire boar spermatozoa and three proteins (ODF2, PRDX5, and AKAP4) were highly phosphorylated in Duroc boar spermatozoa. Most of these proteins belonged to the motile cilium, sperm flagellum, or sperm fibrous sheath components. For example, AKAPs are related to sperm fertility as a platform to integrate the cAMP signaling pathway and others through the binding with ion channels, protein kinases, and small GTP-binding proteins (Skroblin et al., 2010). The phosphorylation motif analysis demonstrated that AKAP4 is the potential substrate of MAPK and PKC protein kinases. This is consistent with the fact that AKAP4 can act as an important regulator between the cAMP/PKA and PKC/ERK1/2 signal pathways in spermatozoa to regulate acrosome reaction and sperm capacitation (Rahamim Ben-Navi et al., 2016). These data confirmed that variation in phosphorylation between Duroc and Yorkshire boar spermatozoa may play a crucial role in the difference in sperm motion and capacitation of two breeds *via* the phosphorylation-mediated signal pathway.

### Classification of Proteins Identified From the DEPs in Duroc and Yorkshire Spermatozoa

At the total protein level, 150/1,697 (∼8.83%) DEPs were identified between Yorkshire versus Duroc pig spermatozoa (fold change ≥ 1.3 and *p* < 0.05) (Supplementary Data 4). Furthermore, the change in expression of most identified protein species (86/150, 57.33%) in the Yorkshire versus Duroc spermatozoa was ±1.30- to ±1.49-fold, whereas only 46 and 18 protein species showed fold change between ±1.50 and ±1.99 and ≥2.00- or ≤2.00-fold in these two breeds, respectively (Figure 2C). As a result, 150 DEPs containing 74 downregulated proteins and 76 upregulated proteins were found in Yorkshire pigs and used for subsequent function analysis and selected validation experiments. The protein with the strongest downregulation in Yorkshire boar sperm was ATP synthase ATP8 (UniProt Accession No. B6EDV3) (Supplementary Data 4). The proteins with the strongest decrease in Yorkshire boar sperm further include signaling and sperm motility proteins (AKAP4, ODF1, WGA16) implicated in fertility regulation, and adhesive glycoprotein (THBS1). On the other hand, the most increased proteins in Yorkshire boar sperm (Supplementary Data 4) also surprisingly comprised the mitochondrial membrane ATP synthase ATP8 (UniProt Accession No. A0A076EBU5) but had a different UniProt accession. The extreme expression difference of the same protein between the two breeds is probably due to the mutation of ATP8. Further analysis of the unique peptide segment of identification ATP8 also found that there were two amino acid differences between B6EDV3 (IYLPLLLPPR, high expression in Duroc boar sperm) and A0A076EBU5 (IYLPLSLPLR, high expression in Yorkshire boar sperm). The list of upregulated proteins also comprises proteins involved in glycometabolism (GK, LDHC); and proteins implicated in ATP binding (RUVBL1, RUVBL2).

**FIGURE 6:**
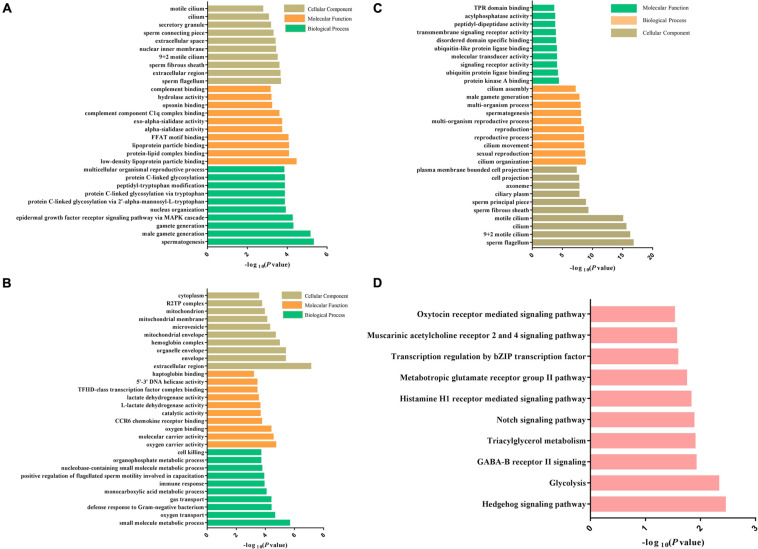
Bioinformatics analysis of the DEPs and DPPs between Yorkshire and Duroc boar Spermatozoa. **(A)** Downregulated DEP GO terms. **(B)** Upregulated DEP GO terms. **(C)** GO enrichment analysis of the DEPs between Yorkshire and Duroc boar spermatozoa. **(D)** The top 12 KEGG pathways in the DPPs between Yorkshire and Duroc boar spermatozoa.

The DEPs between Duroc and Yorkshire spermatozoa were functionally annotated against the UniProt databases and then grouped based on GO enrichment: biological process, biological functions, and cellular component. Between the two breeds, GO analysis revealed that downregulated proteins were enriched in the biological process categories of the reproduction process, such as spermatogenesis, male gamete generation, and multicellular organism reproduction. For the molecular function, most groups were related to the oxygen activity, molecular carrier activity, oxygen binding, catalytic activity, and L-lactate dehydrogenase activity. For the cellular component, the downregulated proteins were enriched in sperm fibrous sheath, sperm flagellum, sperm-connecting piece, motile cilium, and secretory granule (Figure 6A). However, for the upregulated proteins, most of the groups were enriched in the cellular component of the extracellular region, organelle envelope, hemoglobin complex and mitochondrial membrane, no-sperm fibrous, and motile cilium. For the biological process and molecular function, most of the enrichment terms were transport process and binding activity, respectively, which were different from the downregulated proteins (Figure 6B). Additionally, it was noted that terms of defense response to fertilization, bacterium, and detoxification enriched in the biological process only for the upregulated proteins. These results suggested that the DEPs might play roles in spermatozoa motility.

### Differentially Accumulated Phosphopeptides in Duroc and Yorkshire Spermatozoa

We analyzed the phosphopeptide distribution of the fold-change ratio (Yorkshire vs. Duroc), and the cutoff used for identification of the DEPPs was set at 1.3-fold change. In total, we identified 283/1,140 (∼24.82%) phosphopeptides that experienced differential phosphorylation in the two breeds (*p* < 0.05). Among these peptides, 100 increased and 183 decreased in the Yorkshire group, respectively (Figure 2D and Supplementary Data 5). In addition, 310 phosphorylation events were identified from the 283 phosphopeptides, and of these phosphorylation residues, 277 (89.4%) were serine and 33 (10.6%) were threonine (Figure 4B). Among these phosphorylated events, a remarkable bias was identified for the serine residue with a high percentage of ∼89% for all differentially phosphorylated sites. Subsequently, the threonine residue was the second common phosphorylated target, with no phosphotyrosine site being identified as differential phosphorylation in our analysis. In general, we showed a trend for proportionally more peptides undergoing increased, as opposed to reduced, phosphorylation in Duroc versus Yorkshire boar spermatozoa (183 vs. 100, respectively; Table 1).

**TABLE 1:** The list of the differentially expressed phosphoproteins involving the sperm fertility.

No.	Protein accessions	Gene name	Protein description	Phosphorylation site	Abundance alteration	Putative reproduction-related function
1	K7GRU7	ACE	Angiotensin I converting enzyme (peptidyl-dipeptidase A) 1	Ser607, Ser607	Up	Fertilization
2	A0A0B8RW53	ACLY	ATP-citrate synthase	Ser455	Up	Energy production
3	A0A286ZKT4	ALMS1	Alstrom syndrome protein 1	Ser56/Thr57, Ser1969	Up	Sperm motility
4	F1SP29	ACTL7A	Actin-like protein 7A	Ser54	Up	Fertilization
5	P24540	ACYP1	Acylphosphatase-1	Ser95	Up	Unknown
6	F1S498/A0A286ZYY7	ADAM20	Disintegrin and metalloproteinase domain-containing protein 20-like	Thr739/Ser742	Up	Sperm maturation and fertilization
7	F1SPH1	PA2G4	Proliferation-associated protein 2G4	Thr328	Up	Signaling
8	F1RNX2	PDCD5	Programmed cell death protein 5	Ser119	Up	Sperm apoptosis
9	I3LN27	PDZD9	PDZ domain-containing protein 9	Thr142/Ser143/Ser145	Up	Unknown
10	F1SBB1	CABYR-1	Calcium-binding tyrosine phosphorylation-regulated transcript variant 1	Ser125/Ser132/Thr138, Ser125/Ser132, Ser336/Ser340, Ser353/Ser356/Ser358	Up	Sperm motility
11	I3LK18	PKD2L2	Polycystic kidney disease 2-like 2	Ser518	Up	Spermatogenesis
12	B4DCU2	CALM1	Calmodulin 1	Ser10	Up	Sperm capacitation
13	I3LCU9	CARHSP1	Calcium regulated heat stable protein 1	Ser29/Ser31	Up	Unknown
14	I3LGU2	CCDC114	Coiled-coil domain-containing protein 114	Ser553/Ser556	Up	Sperm motility
15	F1SQY0	PPP1R2	Protein phosphatase inhibitor 2	Ser89, Ser89/Ser90	Up	Sperm motility
16	F1SI09	PPP1R7	Protein phosphatase 1 regulatory subunit 7	Ser44/Ser48	Up	Sperm motility
17	F1S598	CCDC151	Coiled-coil domain-containing protein 151	Ser586,	Up	Sperm motility
18	A0A286ZWC2	CCDC7	Coiled-coil domain-containing protein 7	Ser297	Up	Sperm motility
19	C1PIG4	PRKAR2A	cAMP-dependent protein kinase regulatory subunit type II alpha	Ser56/Ser58	Up	Sperm motility
20	A0A287AF38	PROCA1	Protein PROCA1	Ser349, Ser340/Ser349, Ser99	Up	Unknown
21	F1RJW3	CFAP45	Cilia- and flagella-associated protein 45	Ser40, Ser59	Up	Unknown
22	F1SSL6	PSMA3	Proteasome subunit alpha type 3	Ser250	Up	Protein degradation
23	F1S227	PTRH2	Peptidyl-tRNA hydrolase 2, mitochondrial	Ser46	Up	Sperm apoptosis
24	A0A287AR56	QSOX2	Sulfhydryl oxidase 2	Ser596	Up	Sperm maturation
25	I3LC61	CYCL2	Cylicin-2	Thr213/Ser215	Up	Spermatogenesis
26	F1S1R1	CYLC1	Cylicin 1	Ser374/Thr375/Ser379, Ser275	Up	Spermatogenesis
27	I3LNF2	DNAH1	Dynein heavy chain 1, axonemal	Ser66/Thr71	Up	Sperm motility
28	A0A287ASX5	RSPH3	Radial spoke head protein 3	Ser344, Ser288	Up	Sperm motility
29	A0A287AGL6	RSPH6A	Radial spoke head protein 4 homolog A	Ser186	Up	Sperm motility
30	A0A287AL46	SLC26A8	Testis anion transporter 1	Ser617/Ser619	Up	Detoxification
31	F1SJU0	EFCAB6	EF-hand calcium-binding domain-containing protein 6	Ser58/Ser61/Ser62, Ser58/Ser62, Ser48, Ser48/Ser53	Up	Fertilization
32	A0A287AHI3	PPP6R3	Serine/threonine-protein phosphatase 6 regulatory subunit 3	Ser455	Up	Unknown
33	A0A286ZY74	TMEM159	Transmembrane protein 159	Ser21	Up	Fertilization
34	A0A287B7U4	TMEM190	Transmembrane protein 190	Ser127, Ser123/Ser127, Ser123/Thr125/Ser127	Up	Fertilization
35	F1SI89	TMEM202	Transmembrane protein 202	Ser111, Ser107, Ser107/Ser111, Ser98/Ser107/Ser111	Up	Fertilization
36	F1S7B1	SPA17	Sperm surface protein Sp17	Ser141	Up	Spermatogenesis, sperm–egg interaction, sperm capacitation, acrosome reaction
37	D5K8A9	SPACA1	Sperm acrosome membrane-associated protein 1	Thr273/Ser278, Ser291, Ser256, Ser278, Ser256/Thr257, Ser272/Thr273/Ser291	Up	Fertilization
38	A0A287AW88	SPAG9	Sperm associated antigen 9	Ser725/Ser728	Up	Fertilization
39	F1SB33	SYPL1	Synaptophysin-like protein 1	Ser2	Up	Unknown
40	F1SUB4	TBATA	Thymus, brain and testes-associated protein	Ser332/Ser333	Up	Unknown
41	O02705	HSP90AA1	Heat shock protein HSP 90-alpha	Ser263	Up	Protein folding
42	D5K8A4	SPATA3	Spermatogenesis associated 3	Ser148, Ser102/Thr105, Ser199	Up	Spermiogenesis
43	F1RG35	STUB1	STIP1 homology and U-box containing protein 1	Ser25	Up	Protein degradation
44	F2Z5I8	RAB8A	Ras-related protein rab-8a	Ser214	Up	Signaling
45	F1RR24	SPATA32	Spermatogenesis-associated protein 32	Ser136/Ser139, Ser136	Up	Spermiogenesis
46	F1S0P1	RGS22	Regulator of G-protein signaling 22	Ser1250	Up	Signaling
47	A0A286ZJB1	St6galnac2	Alpha-*N*-acetylgalactosaminide alpha-2,6-sialyltransferase 2	Ser43/Thr47, Ser43/Thr47/Ser49, Thr76, Ser95/Ser9, Thr76/Thr79/Ser80, Thr76/Thr79, Ser846	Up	Unknown
48	I3LL86	LRRC37AB	LRRC37AB_C domain-containing protein	Ser513, Ser516, Ser516/Ser518	Up	Unknown
49	F1S2N5	LRRC74A	Leucine-rich repeat-containing protein 74A	Ser73/Thr76	up	Unknown
50	F1RQ56	TSBP1	Testis expressed basic protein 1	Thr317Ser323	Up	Unknown
51	I3LD34	MS4A14	Membrane spanning 4-domains A14	Ser442/Ser451, Ser302/Thr305	Up	Signaling
52	A0A287A852	N/A	Uncharacterized protein	Ser32	Up	Unknown
53	F1S2Q4	NEK9	Serine/threonine-protein kinase Nek9	Ser682	Up	Spermiogenesis
54	F1SAE4	NME8	Thioredoxin domain-containing protein 3	Ser162/Ser166	Up	Sperm tail maturation
55	F1SCU0	NT5C1B	Cytosolic 5’-nucleotidase 1B	Ser67/Ser69/Ser71, Thr66/Ser67/Ser69, Ser69, Ser44, Thr41/Ser44, Ser54, Ser153, Ser19/Ser22, Ser228, Ser40/Ser44, Ser117/Thr120/Ser125, Ser149/Ser153, Ser64/Ser67, Thr72/Ser76, Ser76	Up	Sperm capacitation
56	A0A2C9F3G2	UBC	Polyubiquitin-C	Thr133	Up	Protein degradation
57	A0A287B7D9	EFCAB5	EF-hand calcium-binding domain-containing protein 5	Ser266	Up	Fertilization
58	I3LJA4	ACTL11	Actin-like 11	Ser481, Ser822, Ser85/Ser93, Ser171, Ser263, Ser528, Ser399/Ser402, Ser152, Ser402, Ser528/Thr633, Ser85	Down	
59	A0A286ZQX0	AKAP3	A-kinase anchor protein 3	Thr205/Ser216, Ser186, Ser33, Ser449/Ser457, Ser636, Ser817, Ser449, Ser186, Ser826, Ser700, Ser770	Down	Sperm motility
60	F1RW21/A0A286ZWH7	AKAP4	A-kinase anchor protein 4	Ser206, Ser543, Ser348, Ser815, Ser461, Ser326, Ser386, Ser634, Ser710, Ser470, Ser111, Ser232, Ser526, Ser599, Ser451, Thr199/Ser206, Ser102, Ser136/Ser198/Thr199, Ser250, Ser213, Ser313, Ser136/Ser140, Ser278, Thr199, Ser751/Ser778, Ser451/Ser453, Ser756, Ser336, Ser54, Ser278/Ser289, Ser36, Ser188,	Down	Sperm motility
				Ser198/Thr199/Ser204, Ser136, Ser138, Thr188/Thr199, Ser136/Ser140/Ser141, Ser543/Ser546, Ser213/Ser219, Thr186, Ser274/Ser278		
61	F1SMN4	CCDC136	Coiled-coil domain-containing protein 136	Ser642, Ser454/Ser461, Thr537, Ser517, Ser513	Down	Sperm motility
62	F1RR82	ODF2	Outer dense fiber protein 2	Ser139, Ser95/Ser96, Thr92/Ser95, Ser37, Ser632, Ser129, Ser74	Down	Sperm motility
63	I6R469	CABYR-3	Calcium binding tyrosine-(Y)-phosphorylation regulated transcript variant 3	Thr246	Down	Sperm motility
64	F1S598	CCDC151	Coiled-coil domain-containing protein 151	Ser330	Down	Sperm motility
65	I3LQX1	CCDC96	Coiled-coil domain-containing protein 96	Ser32, Ser36	Down	Sperm motility
66	F1ST87	CCIN	Calicin	Ser229	Down	Spermiogenesis
67	F1SKP4	PLA2G6	85/88 kDa calcium-independent phospholipase A2	Ser12/Ser13	Down	Sperm motility
68	F1SQZ0	PLCZ1	Phosphoinositide phospholipase C	Ser376	Down	Spermiogenesis
69	C3VMK5	CLDN8	Claudin	Thr86, Thr97	Down	Unknown
70	F1RPZ6	CRISP2	Cysteine-rich secretory protein 2	Ser53	Down	Sperm-egg fusion
71	I3LTK6	OAZ3	Ornithine decarboxylase antizyme 3	Ser9/Ser12	Down	Spermiogenesis
72	F1RQP0	PRDX5	Peroxiredoxin 5	Thr45/Thr51	Down	Detoxification
73	A0A287AKY1	PRKAR1A	cAMP-dependent protein kinase type I-alpha regulatory subunit	Ser374	Down	Sperm motility
74	F1RHJ8	DBIL5	Diazepam-binding inhibitor-like 5	Ser46	Down	Sperm motility
75	F1S3K9	PSEN2	Presenilin	Ser365/Ser367	Down	Signaling
76	A0A287AHL5	DNAH7	Dynein heavy chain 7, axonemal	Ser135	Down	Sperm motility
77	A0A286ZTR0	DRC1	Dynein regulatory complex protein 1	Ser454	Down	Sperm motility
78	F1S8J6	RAB2B	Ras-related protein Rab-2B	Ser194/Ser202	Down	Signaling
79	Q29077	ODF1	Outer dense fiber protein 1	Ser32, Ser122/Ser123, Ser194, Ser122, Ser167, Ser86	Down	Sperm motility
80	A0A287BEZ2	RANBP17	RAN binding protein 17	Ser40	Down	Spermatogenesis
81	I3LT05	ROPN1	Ropporin-1	Ser62	Down	Spermatogenesis, sperm motility
82	I3L912	FAM205A	Protein FAM205A	Ser173, Ser80, Ser93, Ser126	Down	RNA biogenesis
83	A0A287AEH4	FAM205C	Family with sequence similarity 205 member C	Ser28, Ser22	Down	RNA biogenesis
84	F1S2W0	FAM71A	Family with sequence similarity 71, member A	Thr115, Ser399/Ser405, Ser182, Ser405	Down	RNA biogenesis
85	F1RQD7	FAM71B	Family with sequence similarity 71, member B	Ser184, Ser518, Ser494, Ser181/Ser183/Ser184, Ser494/Ser495, Ser183/Ser184, Ser24	Down	RNA biogenesis
86	A0A286ZY95	FN1	Fibronectin	Ser2378	Down	Sperm maturation
87	F1RYK8	FSIP2	Fibrous sheath-interacting protein 2	Ser5985/Ser5989, Ser5378, Ser6144/Ser6157, Ser6324, Ser6608, Ser6305, Ser5577, Ser6223, Ser5780, Ser1320, Ser5770, Ser6069/Ser6075, Ser3603, Ser6159, Ser4611, Ser1504/Ser1505, Ser4769, Ser6069, Ser1441	Down	Spermatogenesis, sperm motility
88	F1S605	GSTM3	Glutathione *S*-transferase	Ser204, Ser6, Ser77	Down	Detoxification
89	A0A287A1G6	HK1	Hexokinase-1	Ser871	Down	Energy production
90	Q5S233	SMCP	Mitochondrial associated cysteine-rich protein	Ser87, Ser92, Thr81/Ser87	Down	Sperm motility
91	I3LFS9	IQCF5	IQ domain-containing protein F5	Ser10	Down	Unknown
92	A0A287AI93	IQCN	IQ motif containing N	Ser344, Ser189/Thr191	Down	Unknown
93	F1SGA5	KLHL24	Kelch-like family member 24	Ser88	Down	Protein degradation
94	A0A287BDE3	LIPE	Hormone-sensitive lipase	Ser647	Down	Energy production
95	I3LC15	LOC100515049	Angiotensin-converting enzyme-like	Ser673, Ser688	Down	Spermatogenesis
96	A0A286ZK47	LOC100626097	Fibrous sheath-interacting protein 2-like	Ser912, Thr6543/Ser6544, Ser727, Ser1594/Ser1596, Ser1366	Down	Sperm motility
97	F1SE68	SPATA18	Spermatogenesis associated 18	Ser101, Ser532, Ser10, Ser159, Ser244, Ser13, Ser312, Ser10/Ser13, Ser552/Ser554/Ser556, Ser259/Ser261, Ser296/Ser298/Ser300/Ser302, Ser534, Ser302, Ser300/Ser302, Ser534/Ser536	Down	Spermatogenesis
98	I3LDJ2	SPATA19	Spermatogenesis associated 19	Ser116	Down	Spermatogenesis
99	A0A286ZU97	MTCH2	Mitochondrial carrier 2	Ser106, Ser114	Down	Energy production
100	A0A287BJP9	SPATA31D1	Spermatogenesis-associated protein 31D1-like	Thr329, Thr329/Ser333	Down	Spermatogenesis
101	F1S6E2	SPATA6	Spermatogenesis-associated protein 6	Ser238	Down	Spermatogenesis
102	A0A2K6ANY4	SPEM3	Spem1 domain-containing protein	Ser783, Ser648, Ser1105	Down	Unknown
103	A0A286ZZH0	SSMEM1	Serine-rich single-pass membrane protein 1	Ser54	Down	Sperm motility
104	F1SIC8	TBC1D21	TBC1 domain family member 21	Ser15	Down	Sperm motility
105	A0A286ZJB1	St6galnac2	Alpha-*N*-acetylgalactosaminide alpha-2,6-sialyltransferase 2	Thr146	Down	Unknown
106	A0A288CFT0	TPI1	Triosephosphate isomerase 1	Ser35	Down	Energy production
107	A0A288CG60	SUN5	SUN domain-containing protein 5	Ser46	Down	Fertilization
108	A0A286ZM55	MROH1	Maestro heat-like repeat family member 1	Ser7	Down	Unknown
109	F1STE2	TSGA10	Testis-specific protein 10 protein	Ser687	Down	Fertilization
110	I3L5F8	TXNDC15	Thioredoxin domain-containing protein 15	Ser117	Down	Sperm motility
111	A0A287BBB5	VAPA	Vesicle-associated membrane protein-associated protein A	Ser209/Ser211	Down	Unknown
112	F1S2F6	VDAC2	Voltage-dependent anion-selective channel protein 2	Ser115	Down	Energy production
113	A0A286ZKT4	ALMS1	Alstrom syndrome protein 1	Ser756/Ser765	Down	Unknown
114	A0A287AF38	PROCA1	Protein PROCA1	Ser99	Down	Unknown

The 283 phosphopeptides came from 102 phosphoproteins; thus, these phosphoproteins were regarded as DPPs. Of these DPPs, 65 held only one DEPP, 12 held two DEPPs, and 25 held three or more DEPPs (Supplementary Data 5). Thus, 39 DPPs were identified as being targeted for multiple phosphorylated sites, with 10 most prominent examples being AKAP4, FSIP2, SPATA18, NT5C1B, ACTL11, AKAP3, ST6GALNAC2, FAM71B, ODF2, and SPACA1, each with as many as 41, 18, 15, 15, 11, 11, 8, 8, 6, and 6 DEPPs, respectively (Supplementary Data 5). The DPPs of the top 10 DEPPs with multiple phosphorylation events accounted for almost half of all DPPs. The greatest differences among the regulated events were seen for the Ser513 and Ser517 of CCDC136, the Ser2378 of FN1, the Ser1441 of FSIP2, the Ser206, Ser348, Ser543, and Ser826 of AKAP4, the Ser300 and Ser302 of SPATA18, the Ser126 of FAM205A, and the Ser117 of TXNDC presenting fold changes > 3. Based on the expression pattern of the phosphorylated peptides, 100 increased phosphopeptides belong to 54 phosphoproteins and 183 decreased phosphopeptides belong to 52 phosphoproteins (Figure 2D). Four phosphoproteins (e.g., CCDC151, ALMS1, PROCA1, and ST6GALNAC2) containing both upregulated and downregulated unique phosphopeptides (Supplementary Data 5) were counted as both increased and decreased proteins for the subsequent GO analysis. To compare the number of DEPs and DPPs, 11 downregulated proteins (e.g., SMCP, ODF1, AKAP4, AKAP3, FAM714D, SPEM3, SUN5) and 1 upregulated protein (SPATA3) were overlapped respectively (Figures 5B,C). However, 71 out of a total 110 DPPs (∼69.1%) were not found in the DEPs but found in other proteins with no significant difference changes (Figure 5D). For example, ROPN1, involved in fibrous sheath integrity and sperm motility (Fiedler et al., 2013), was found to have ∼2 times higher phosphorylation expression in Duroc spermatozoa in the Ser62 site, while there was no significant change in the protein level between the two breeds of sperm. SPACA1, a testis-specific expression gene, is localized in sperm acrosomes and is found to be important for sperm–egg binding and fusion (Fujihara et al., 2012; Yamatoya et al., 2019), which had multiple higher phosphorylation sites (e.g., Ser291, Ser278, Ser256, Thr273/Ser278) in Yorkshire boar sperm. These results suggested that a high percentage of phosphorylated proteins in boar spermatozoa are probably associated with a breed difference in regulating sperm function.

**FIGURE 7:**
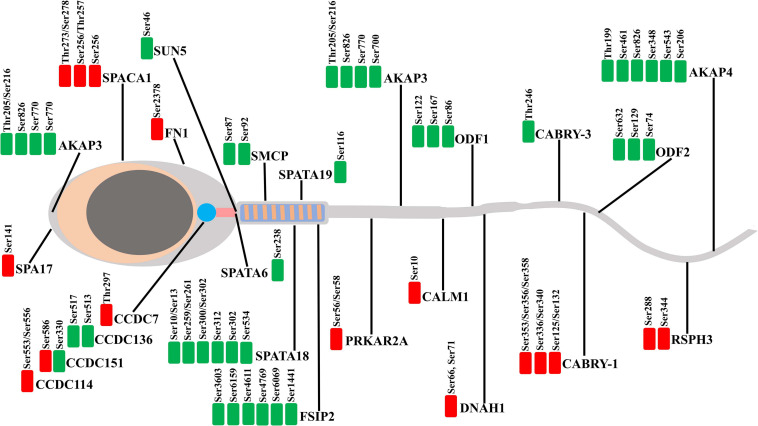
Illustration gathering the localization of the proteins belonging to the DEPPs between Yorkshire and Duroc boar spermatozoa. The downregulated and upregulated phosphorylated sites shown in the green and red plots, respectively.

To understand the biological roles of differential protein phosphorylation in different genetic backgrounds in boar spermatozoa, the DPPs were annotated by the GO term enrichment and KEGG pathway analysis. First, GO enrichment for all DPPs was conducted, and 48 GO categories were enriched. As shown in Figure 6C, the DPPs were clustered into top GO terms depending on their biological processes, including cilium organization, sexual reproduction, cilium movement, spermatogenesis, cilium assembly, and multiorganism process. The DPPs were classified into top groups based on their cell component, and these GO terms contained sperm flagellum, motile cilium, sperm fibrous sheath, sperm principal piece, and ciliary plasm, and axoneme. Based on their molecular function, the DPPs were classified into 10 groups including protein kinase A binding, ubiquitin protein ligase binding, TPR domain binding, transmembrane signaling receptor activity, molecular transducer activity, acylphosphatase activity. These data of GO term enrichment dramatically suggest that genetic background interrelated with changes in the phosphorylation levels of different sperm-specific proteins primarily involved in sperm fertility regulation (Figure 7). Among them, we saw both upregulated and downregulated DPPs related to sperm motility, sperm capacitation, and acrosome reaction such as AKAP3, AKAP4, ODF1, FSIP2, and ODF2, the proteins of the coiled-coil domain-containing protein family (CCDC136, CCDC114, CCDC7, and CCDC151), SPACA1, DNAH1, SPA17, and CABYR-1. These results suggested that regulation of boar sperm activity is tightly coupled with the opposing action of cellular kinases and phosphatases.

The 110 DPPs were further analyzed using the KEGG database. In total, we found 10 enriched KEGG pathways in different breeds. As illustrated in Figure 6D, many fundamental biological pathways were overrepresented by phosphoproteins identified in this study, including hedgehog signaling pathway, glycolysis, triacylglycerol metabolism, Notch signaling pathway, and oxytocin receptor-mediated signaling pathway.

### Phosphoproteome Integration in a Molecular Network

**FIGURE 8:**
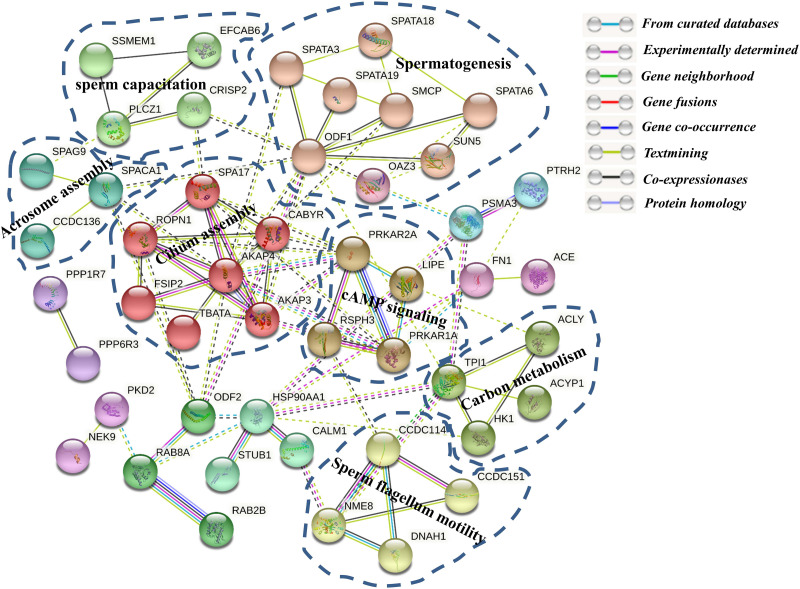
The protein–protein interaction (PPI) network of the differentially phosphorylated phosphoproteins between Yorkshire and Duroc boar spermatozoa. The functional classification of the DPP group is shown in bold.

To further investigate the relationships among these phosphoproteins, the STRING database (see footnote 4) was used to identify protein–protein interactions (PPI) and to construct a network of interactions based on a variety of sources including various interaction databases, genetic interactions, text mining, and shared pathway interactions. The DPPs were uploaded for the PPI network construction using MCL clustering (inflation parameter = 3), and interactions with at least medium confidence were set by default (interaction score >0.4). As shown in Figure 8, **51** proteins relate to 184 paired relationships. At a glance, the network is organized around several strongly connected subnetworks, the majority of which are directly associated with sperm capacitation and sperm motility. Firstly, we identified a spermatogenesis-related subnetwork, which is composed mainly of upregulated proteins in Duroc boar sperms (e.g., SPATA18, SPATA6, SPATA19, SMCP, ODF1, OAZ3, and SUN5) (Figure 8). It contains SPATA18, multiply hyper-phosphorylated in Duroc boar sperm, which is a crucial regulator of mitochondrial quality that participates in repair or degradation of the injured mitochondria (Kitamura et al., 2011). Indeed, it was shown that as a testis-associated p53 target gene, SPATA18 can serve as a monitor of sperm cell differentiation and play a crucial role in the maturation of spermatids into spermatozoa, and proposed to be a structural component of the sperm flagella (Bornstein et al., 2011). Recently, a research in humans demonstrated that the attenuated levels of SPATA18 reduce fertility caused by defects in sperm development (Bornstein et al., 2011). To date, there is no report concerning SPATA18 phosphorylation in boar sperm. Another interesting member of this subnetwork is SPATA3, of which protein products might be involved in spermatogenesis regulation, mainly in spermatogenesis cell apoptosis or spermatogenesis (Ota et al., 2004; Rolland et al., 2014). Seven calcium assembly related DPPs form another subnet including SPA17, FSIP, CABYR, AKAP3, AKAP4, TBATA, and ROPN1. AKAP4, a major sperm fibrous sheath protein that localizes to the entire length of the flagellum in rodent and mammal spermatozoa, is playing multiple roles in flagellar structure, chemotaxis, capacitation, sperm motility, and regulation of signal transduction pathways (Jumeau et al., 2018; Blommaert et al., 2019). AKAP4 is annotated to interact with six other proteins, SPA17, FSIP, CABYR, AKAP3, TBATA, and ROPN1. Intriguingly, we found that CABYR was both hyper- and hypo-phosphorylated with different protein isoforms in Yorkshire boar sperms. This protein is a testis-specific phosphoprotein located in the sperm flagella and regulated by phosphorylation during sperm capacitation (Pelloni et al., 2018). In addition, CABYR can interact with AKAPs in the fibrous sheath of the sperm flagella by its RIIα domain and serve as scaffold and calcium carrier for the enzyme complexes, which mediate energy production leading to hyperactivation of the sperm (Naaby-Hansen et al., 2002; Li et al., 2011; Young et al., 2016). The third largest subnet is composed of five DPPs involved in sperm flagellum motility, including CCDC114, CCDC151, DRC1, NME8, and DNAH1. The other subnets in this PPI network were associated with carbon metabolism, sperm capacitation, and cAMP signaling.

Furthermore, A STRING protein network consisting of 12 overlapped proteins between DEPs and DEPPs of interest in this study is shown in Figure 5E. The results indicated that the proteins, such as ODF1 and SMCP, interacted with six other proteins. Among these interactions, ODF1, as the molecular focus, is involved in sperm motility and flagellum development (Mariappa et al., 2010; Hetherington et al., 2017). In this network, only STAPA3 was upregulated in Yorkshire boar sperm.

### Confirmation of DEPs by Western Blot Analysis

**FIGURE 9:**
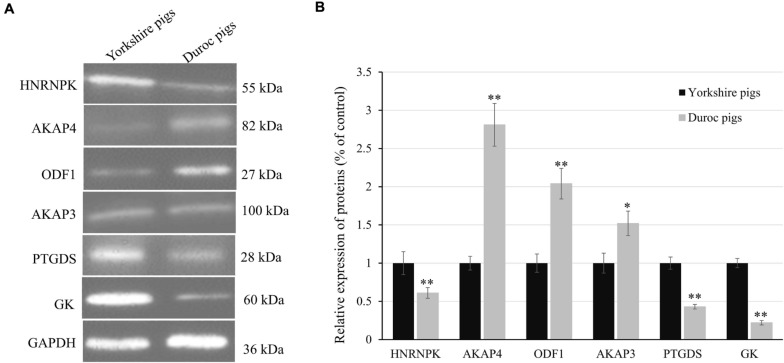
Confirmation of differential expression of proteins between Yorkshire and Duroc boar spermatozoa. **(A)** Expression levels of HNRNPK, AKAP4, ODF1, AKAP3, PTGDS, and GK in Yorkshire and Duroc boar spermatozoa were confirmed by Western blotting. GAPDH served as a loading control. **(B)** Quantified relative band intensity ratio of **(A)**. *p < 0.05, **p < 0.01.

Proteins with significant fold change and of interest based on their biological function were validated by Western blot analysis. This analysis also included proteins exhibiting the consistent trend of upregulating expression in Yorkshire breed (e.g., HNRNPK, PTGDS, and GK), as well as those that had a higher abundance in Duroc spermatozoa (e.g., AKPK4, AKAP3, and ODF1). All Western blot experiments were performed in triplicate using pooled biological samples (*n* = 4 sample) differing from those employed for iTRAQ proteome analyses and, in each experiment, GAPDH (glyceraldehyde-3-phosphate dehydrogenase) acted as an endogenous control to normalize the targeted proteins (Figures 9A,B and Supplementary Figure 3). The results confirmed the differential expression of the seven spermatozoa proteins between two breeds, with each of these proteins’ expression closely paralleling the trends identified by MS analyses (Figure 8B). Accordingly, a linear regression comparing the fold changes recorded for each of these targets revealed a significant correlation (*R*^2^ = 0.98; *p* < 0.01) between the quantification data obtained *via* immunoblotting analyses. Taken together, such findings support the accuracy of our data in reflecting the spatial patterns of porcine spermatozoa proteomic signatures.

## Discussion

In this study, we provide a first comprehensive quantitative analysis of the protein and protein phosphorylation differences in sperm between Yorkshire and Duroc boar. The iTRAQ-based proteomics and phosphoproteomics strategy is the most powerful technique for the global analysis of signaling networks in defined biological systems (Macek et al., 2009; Deracinois et al., 2013). In the field of livestock reproduction, proteomics has been used to illuminate the molecular basis of sperm freezability, sperm motility, or fertility (He et al., 2016, 2019; Maciel et al., 2018; Pini et al., 2018; Kasimanickam et al., 2019; Perez-Patino et al., 2019a, b; Peris-Frau et al., 2019). However, an in-depth study of proteome-combined phosphoproteome determining breed differences in boar sperm was not conducted so far. Here, we have performed a global analysis of quantitative proteomics coupled with phosphopeptide-enrichment strategies to unravel the boar spermatozoa proteome and identify signatures of PTM associated with the breed differences. The current workflow led us to identify 150 sperm proteins and 283 sperm phosphosites with a dramatically altered abundance in sperm because of the breed difference with sperm motility and capacitation, fertility.

### Sperm Proteome Differences Between Yorkshire and Duroc Boar

In the present study, an updated proteome of porcine spermatozoa was generated using iTRAQ combined with LC-MS, which identified 1,745 proteins, and 1,738 of them quantified, most of them encoded in the Sus scrofa taxonomy. The total number of identified proteins was similar to the 1,723 proteins and 1,597 proteins identified recently by Xinhong et al. (2018) and Perez-Patino et al. (2019c), respectively, using iTRAQ technology, which was more than 1,157 proteins identified by Perez-Patino et al. (2019b) using the LC-ESI-MS/MS-based SWATH approach but was substantially lower than 2,728 proteins identified by Feugang et al. (2018) using a shotgun strategy. The difference in the number of identified proteins among the iTRAQ, SWATH, and shotgun approaches may be related to the methodological differences used for peptide enrichment detection, and data processing for protein identification and validation. For example, iTRAQ requires high collision energy, resulting in the loss of fragments of sequence information, which is more restrictive in protein identification than the shotgun strategy (Wiese et al., 2007). In addition, ∼2,000 identified proteins of porcine sperm proteome are far less than the ∼7,500-protein pf human spermatozoa proteome (Amaral et al., 2014), which demonstrates the yet incomplete functional and structural annotation of the porcine protein-coding genes, and many proteins still have no function assigned to them.

The distribution of total identified proteins into the GO enrichment showed a similar pattern to that observed in the human, mouse, or bovine sperm proteome (Martinez-Heredia et al., 2006; Chauvin et al., 2012; Kasvandik et al., 2015). The most enriched functions of sperm proteins were related to the metabolic processes, protein and tRNA transport, nuclear organization, or processes related to sperm function, which demonstrates that the central proteomic architecture of sperm is broadly comparable from these mammalian species. Among the proteins, the GO distribution of DEPs differed from the totality of identified proteins, particularly in biological processes and cellular components. Compared to the total identified proteins, the proportion of DEPs related to reproductive processes involved in sperm motility, sperm capacitation, sperm–oocyte binding, and fusion were significantly higher in Duroc boar sperm. Particularly, the cytoskeletal part proteins of sperm, such as SMCP, AKAP3, AKAP4, SUN5, and ODF1, play an important role in sperm function regulations. Because all boars used in the study were produced and raised in the same environment and fed the same ration, differences between the two breeds in sperm proteins were mainly attributed to their genetic background effect. This identification therefore dramatically indicated that the differences in protein abundance between the two breeds with different genetic backgrounds affected the sperm functionality. In general, the cytoskeletal part proteins in Duroc spermatozoa were superior to those of the Yorkshire spermatozoa and supplied a better condition for the physiological characteristics and structure stability of Duroc boar sperm.

The DEPs resulting from the two different breeds of sperm could help us understand the proteins influencing sperm functionality of genetic factors and provide further insight into their functions. A total of 150 proteins exhibited significantly different expression patterns between the two groups, and 145 proteins were successfully identified as characterized proteins. We determined that most of these proteins were involved in regulation of the reproductive process by bioinformatics analysis. More importantly, 20–25 of these proteins were increased in the Duroc boar spermatozoa, which were suggested to participate in the pathway of spermatogenesis, fertilization, or sperm motility and capacitation, based on their changing expression patterns in Duroc sperm compared to Yorkshire sperm. The changed expression of the above proteins might contribute to the sperm activity, capacitation, and sperm–egg interaction in Duroc boars.

Sperm proteins directly related to the reproductive function play a key role in the fertilization process. For instance, AKAPs are a group of evolutionarily conserved regulators, necessary for sperm motility, sperm capacitation, and acrosome reaction, which have the widespread function of binding to the regulatory subunit of cAMP-PKA and directing the kinase holoenzyme to particular subcellular compartments (Vizel et al., 2015; Autenrieth et al., 2016). AKAP4 binds with AKAP3; they are two major components of the sperm sheath responding to the regulation of the metabolic pathways and signal transduction that maintain sperm function (Brown et al., 2003; Nixon et al., 2019a). AKAP4 plays a key role in completing fibrous sheath assembly, whereas AKAP3 is involved in organizing the basic structure of the fibrous sheath. Previous studies have shown that absence or weak expression of AKAP3 and AKAP4 has been described over the years as being related to sperm dysfunctions with motility impairments (Miki et al., 2002; Hillman et al., 2013; Xu et al., 2020). In contrast, the upregulation of either AKAP4 or AKAP3 in frozen-thawed boar sperm might be associated with their premature capacitation (Chen et al., 2014; Perez-Patino et al., 2019b). A higher expression of AKAP3 and AKAP4 in Duroc boar sperm might promote the sperm function; they play a crucial role in sperm motility. We also observed many DEPs such as ODF1 (Zhao et al., 2018), SMCP (Nayernia et al., 2002), SUN5 (Shang et al., 2017), PRM2 (Zalata et al., 2016), RAB10 (Lin et al., 2017), and OAZ3 (Tokuhiro et al., 2009), which were more abundant in Duroc sperm than in Yorkshire sperm and have previously been associated with the reproductive efficiency of sperm. ODF1 is the major protein of the outer dense fibers in the mammalian sperm tail, taking part in sperm motility and flagellum development. In ODF1 knockout mice, sperm show a weakness in the connecting piece and a disorganized mitochondrial sheath and are also easily decapitated (Yang et al., 2012). Further studies have shown that the lack of ODF1 is observably reduced within infertile men, correlating a weakness between the head and neck regions of sperm (Hetherington et al., 2016). SMCP is a component of the keratinous capsule surrounding sperm mitochondria and play a key role in the stabilization and organization of the helical structure of sperm sheath (Nayernia et al., 2002). Deletion or reduction of SMCP impaired sperm motility, leading to spermatozoa failing to swim in the female reproductive tract and piercing the egg membranes during fertilization (Nayernia et al., 2002; Huang et al., 2016). A decreased expression of these proteins could indicate a weakening in the tail and/or the mitochondrial sheath of sperm, which would explain why Yorkshire spermatozoa have relatively low motility parameters than Duroc. Similarly, the higher abundance of the other four proteins could also clarify the improved functionality of Duroc sperm. In contrast, PSP-I, PSP-II, AQN3, SERPINE2, and TIMP-2, which were negatively related to sperm reproductive efficiency, were decreased in Duroc sperm compared to Yorkshire. PSP-I, PSP-II, and AQN3 are three major members of the sperm adhesin family that are low-molecular-weight glycoproteins and primarily secreted by the seminal vesicles (Topfer-Petersen et al., 1998; Centurion et al., 2003; Feugang et al., 2018). Once bound to the sperm plasma membrane, sperm adhesins are involved in regulating some of the most relevant sperm functions, such as sperm motility, sperm capacitation, acrosome reaction, or sperm-zona pellucida binding. However, if these proteins are overexpressed in sperm, they may also be harmful to sperm function. For example, the high expression of PSP-I/PSP-II dipolymer demonstrated decapacitating effects in boar sperm, which shows a significantly negative correlation with sperm functionality in liquid-preserved pig AI-semen doses (Caballero et al., 2009; Dyck et al., 2011). Furthermore, sperm adhesins increase with the decrease in sperm concentration, resulting in an increase in sperm adhesion concentration in the semen-poor fraction (Garcia et al., 2009). The higher expression of these sperm adhesins in Yorkshire spermatozoa may be closely related to the lower sperm activity and sperm concentration in Yorkshire boar. In addition, SERPINE2, also known as glia-derived nexin or protease nexin-1, has a wide range of serine protease-specific anti-protease activity that inhibits sperm capacitation by preventing the cholesterol from flowing out of the sperm plasma membranes and inhibiting the increase of sperm protein tyrosine phosphorylation (Lu et al., 2011; Li et al., 2018). Previous studies have also demonstrated that a higher abundance of SERPINE2 was related to lower fertility in modern artificially inseminated sows (Perez-Patino et al., 2019a). TIMP-2 is a specific natural inhibitor for MMP-2 which is a member of matrix metalloproteinases (MMPs) and thought to be associated with sperm motility, sperm capacitation, and fertilization (Robert and Gagnon, 1999; Belardin et al., 2019). Thus, the content and activity of TIMP-2 in sperm showed a significantly negative correlation with fertility. The changed expression pattern of these proteins in the Yorkshire spermatozoa can both impact the reproductive efficiency of Yorkshire boars. Therefore, we suggest that the differences in the expression levels of reproductive efficiency-related proteins may be one of the important reasons for reproductive differentiation between Yorkshire and Duroc sperm.

### Sperm Phosphoproteome Differences Between Yorkshire and Duroc Boar

It is worth noting that since mature sperm are silent in both transcription and translation, their function is highly dependent on the addition of exogenous proteins (e.g., new sperm proteins during epididymal transit) or PTMs to their existing protein complement (Diez-Sanchez et al., 2003; Dun et al., 2012; Porambo et al., 2012). Phosphorylation, as the most important PTM, allows rapid control of the activity of signaling and regulatory proteins, which is essential in the regulation of sperm function (Parte et al., 2012; Porambo et al., 2012; Dacheux and Dacheux, 2014). In recent years, the phosphoproteomics strategy has been widely used to uncover the molecular mechanisms of capacitation, sperm maturation, sperm motility, and infertility in human, mice, or other animals (Ficarro et al., 2003; Wang et al., 2015; Gazo et al., 2017; Castillo et al., 2019; Nixon et al., 2019b; Urizar-Arenaza et al., 2019; Martin-Hidalgo et al., 2020). However, a much deeper analysis of the phospho-regulation in the porcine sperm has not been conducted yet. In this study, we show the first global comprehensive analysis of the phosphoproteome of the porcine sperm with different breeds. Using TiO_2_-based phosphopeptide enrichment combined with LC-MS/MS analysis, we have identified 1,064 phosphopeptides coming from 363 proteins, resulting in the most complete research of the porcine sperm phosphoproteins to date. According to the results reported in this study, phosphorylated proteins represent 19.8% of the porcine sperm proteome and are primarily involved in nuclear pore organization, sperm–egg recognition, cilium- and flagellum-dependent movement, and metabolism. Lately, Urizar-Arenaza et al. (2019) reported the largest description of the human sperm phosphoproteome with 3,500 identified phosphosites belonging to 1,332 proteins. Compared to the number of identified phosphorylated proteins in the human sperm proteome, the number of porcine sperm phosphoproteins is much smaller, which may be related to the incomplete annotation of the pig proteome. In addition, more than 51.5% porcine-phosphorylated proteins were found in human phosphorylated proteomes, and the most prominent phosphoproteins with multiple phosphorylation events were similar. For example, the five most prominent examples in human sperm are FSIP2 (145), AKAP4 (102), AKAP3 (71), CABYR (45), and ODF2 (37) (Urizar-Arenaza et al., 2019), while the five most prominent examples in porcine sperm are AKAP4 (55), FSIP2 (34), ODF2 (33), AKAP3 (34), and ACTL11 (27). Compared to the humans and mice, data on porcine phosphorylation sites are very scarce so far. Therefore, most of the phosphorylation sites identified in the present study are novel protein phosphorylation sites with uncharacterized or unknown functions in porcine sperm. Thus, the investigation of novel phosphorylation events to elucidate the functions of these sperm phosphoproteins will undoubtedly be of great interest for uncovering the complex regulatory mechanisms involved in porcine sperm function and contribute to the functional categorization of poorly annotated porcine proteins.

Analysis of phosphorylated residue distribution in porcine sperm phosphoproteome demonstrates that the proportion of pS, pT, and pY (86.8, 12.3, and 0.9%, respectively) is more closely in approximate with those experienced in vertebrate cells showing that pS, pT, and pY occur at an estimated ratio of 1,000:100:1 (Raggiaschi et al., 2005). However, only 10 tyrosine phosphorylation sites were identified in this study, which are relatively few. It could be interpreted by the fact that we separated the boar semen fractions under non-capacitated conditions. In general, capacitation seems to be a phenomenon specific to mammals, and accumulating evidence indicates that it generally involves a burst of protein-tyrosine phosphorylation (Ijiri et al., 2012). Therefore, tyrosine phosphorylation is usually minimal in uncapable sperm. The proportion of tyrosine phosphorylation in previous studies of uncapable sperm phosphorylation in humans was also roughly equivalent to our results (Urizar-Arenaza et al., 2019; Martin-Hidalgo et al., 2020).

Motif analysis of regulated phosphorylation sites identified through phosphoproteomic datasets is usually used to predict the protein kinases that respond to the phosphorylation. Consistent with previous research (Huang et al., 2018; Yang et al., 2018), our phosphoproteomics results also demonstrated that some crucial spermatogenesis-related proteins (e.g., SPACA1, SPATA18, and SPATA31D1) and sperm motility-related proteins (e.g., AKAP4, CFAP45, ODF2, CABYR, and CDC96) were respectively phosphorylated at PKCs, AKT, GSK-3, CDK, and MAPK motifs, demonstrating that these proteins are potential targets of the corresponding kinases. Considering the identified phosphorylation motifs, combined with the known consensus on phosphorylation site specificity of serine/threonine kinases, we can predict some important target proteins of upstream kinases involved in the spermatogenesis and sperm motility-related pathways that have different regulations in Yorkshire and Duroc boar sperm. For example, the phosphopeptides identified in porcine AKAP4 at Ser136 sites (YALGFQHALSPSASSCK) contain a specific motif pSP for ERK1/2 that enhances the effect of AKAP4 to bind to the type II regulatory subunit of PKA (Miki and Eddy, 1999). Thus, possible AKAP4 activation by ERK1/2 allows us to concatenate ERK1/2 to the upstream cAMP/PKA signal pathway which plays a key role in human sperm activity, sperm capacitation, and acrosome reaction (Rahamim Ben-Navi et al., 2016).

Phosphoproteomic research shows that 283 DEPPs belonging to a total of 102 proteins of porcine sperm are identified at differential amounts between Yorkshire and Duroc boar. Among them, 54 phosphorylated proteins are more abundant in the Yorkshire spermatozoa, whereas 52 phosphorylated proteins have a higher expression in Duroc sperm (Table 1). Significantly, this study showed that the phosphoproteins of Duroc spermatozoa are mainly involved in sperm function and spermatogenesis, such as sperm motility, sperm–egg binding and recognition, and capacitation. These sperm processes are crucial to the fertilization potential in porcine sperm. This is in line with the fact that Duroc sperm also show the best-quality characteristics, being used as a terminal parent in the modern pig industry. Our findings suggest a differential phosphorylated regulation of boar spermatozoa proteins manipulating sperm motility between the different breeds. In particular, the DPPs are primarily involved in crucial requirements of the flagellum for the sperm movement and control of axoneme mechanical components. These results were compared with a similar research in human that the differential regulation of phosphoproteins between high and low motility spermatozoa is mainly associated with cytoskeletal, metabolic, and fibrous sheath proteins (Martin-Hidalgo et al., 2020). In reality, we have identified several more abundant phosphoproteins in Duroc boar sperm which play a role in flagellum assembly and sperm motility (Figure 7), such as FSIP2, ODF2, ODF1, CABRY, SMCP, SPATA18, SPATAT19, SUN5, SLC26A8, DNAH1, AKAP3, AKAP4, coiled-coil domain-containing proteins (CCD7, CCD151, CCD136, CCD114), and cilia- and flagella-associated protein CFAP45. Also, we detected HSP90AA1 which is differentially phosphorylated between Yorkshire and Duroc spermatozoa, sustaining the function of HSPs in porcine male fertility.

In the process of fertilization, sperm not only need to swim to the sites that bind to the egg in the female reproductive tract but also acquire the ability to fertilize with the egg. Therefore, sperm motility is a key factor affecting the fertilization. Usually, sperm motility is mainly formed by the swing of long flagella in the tail of sperm, which is not only affected by the external environment but also regulated by several internal signal pathways (Vernon and Woolley, 2004). The cAMP/PKA signal pathway and Ca^2+^ signal pathway are the two most important signal pathways to regulate mammalian sperm motility (Dey et al., 2019). It is common knowledge that cAMP works directly on PKA, and the specificity and function of PKA in cells are attributed to its localization through anchoring protein AKAPs in response to cAMP signaling. AKAPs take an active part in PKA-dependent protein tyrosine phosphorylation, two members of which also increased the phosphorylation in Duroc boar spermatozoa. As mentioned before, AKAPs serve as scaffolding proteins for integrating the cAMP-PKA pathway and Ca^2+^ signals, and the increased phosphorylation of AKAP3 and AKAP4 could be the connectors between different transduction cascades in sperm motility regulation (Skroblin et al., 2010; Urizar-Arenaza et al., 2019), which strongly supports our research. Similarly, we also detected relative phosphorylation alterations in other proteins known to participate in PKA-dependent pathways including CABYR, ROPN1, CALM1, PRKAR2A, and PRKAR1A. CABYR serves as a key ingredient belonging to the Ca^2+^ signal pathway during the capacitation and acrosome reaction, which performs putative motifs for self-assembly and for binding PRKAR2A (type II regulatory subunit of PKA R-subunit), AKAP3 and AKAP4 (Li et al., 2011; Young et al., 2016). Interestingly, the dephosphorylation of CABYR inhibits its binding capacity with calcium. ROPN1, a capacitation-related protein, is an important ingredient of the fibrous sheath in mammalian sperm, and it resides in the primary piece and the terminal piece of sperm flagella, which is also an important PKA regulator and is active in sperm motility regulation (Chen et al., 2011; Li et al., 2011; Kwon et al., 2014). Another study showed that ROPN1 interacts with AKAP3, and this interaction depends on AKAP3 phosphorylation (Fiedler et al., 2013). These evidences were consistent with our observation of an increased phosphorylation of AKAP3 and AKAP4 in Duroc, indicating that an upregulation of AKAP3 and AKAP4 probably leads to activation of cAMP-mediated PKA signaling. The increased phosphorylation of these proteins suggests an effect on PKA signaling. In addition, PRKAR2A and PRKAR1A are two regulatory subunits of PKA, and PRKAR2A was expressed primarily in the axonemal region of the mammal sperm flagellum, while PRKAR1A was present in connection with sperm’s outer dense fibers and fibrous sheath (Fiedler et al., 2008; D’Amours et al., 2018). PRKAR2A contains a phosphorylation site in the inhibitory domain, whereas PRKAR1A subunits do not; this may result in an altered binding affinity for the catalytic subunits, and both of them dimerize by their N-terminal domains and bind to AKAPs (Wu et al., 2007). In the present study, the upregulated phosphorylation of PRKAR1A (Ser374) and downregulated phosphorylation of PRKAR2A (Ser56/Ser58) were detected in Duroc spermatozoa, respectively, indicating that the different phosphorylation of the two PKA regulatory subunits has different functions in sperm motility. Meanwhile, both of the proteins tend to co-express or interact with AKAP3, AKAP4, and other cytoskeletal proteins such as CABYR, ROPN1, FSIP2, ODF1, ODF2, or SMCP. Therefore, we suggested that the phosphorylation of sperm-specific proteins is involved in the regulation of boar sperm motility mainly through the cAMP/PKA signal pathway in different breeds.

On the other side of the coin, the phosphoproteins abundant in Duroc boar sperm are principally involved in sperm energy metabolism, such as TPI1, HK1, and LIPE, and particularly related to regulation of glycolysis pathway and lipolysis. Generally, sperm motility is directly controlled by energy resources, including glycolysis, lipid metabolism, and oxidative phosphorylation (Williams and Ford, 2001). Previous research showed that phosphorylation of these proteins can promote the energy generation *via* catabolic pathways in all kinds of cells. Given these data, the results showed that the sperm potential fertility in Duroc boar was significantly higher than in Yorkshire pigs. This is broadly in line with the higher sperm motility of Duroc boar. In addition, our phosphoproteomics data also found more phosphorylated abundance of ATP-citrate synthase ACLY that links energy metabolism provided by catabolic pathways to biosynthesis in the Yorkshire sperm fraction. These results are consistent with those from the previous phosphoproteome research of human sperm, which showed that carbohydrate metabolic pathways altered in human sperm with the low-motility group (Martin-Hidalgo et al., 2020). In a word, these results supply a valuable insight into the molecular basis of differences in reproductive efficiency between Duroc and Yorkshire boar spermatozoa.

## Conclusion and Perspectives

In summary, the results of this study fully reveal the multiple changes in the protein levels and phosphorylation status between Yorkshire and Duroc sperm and discussed the relationship between pig reproductive efficiency and fertility ability. Through the parallel and large-scale quantitative analyses of porcine spermatozoa proteome and phosphoproteome, a variety of new molecular mechanisms that may help to understand spermatogenesis, sperm motility, fibrous sheath and cytoskeleton, sperm–egg recognition, and energy metabolism were identified. Investigations of possible phosphokinase interactions have revealed that several regulatory kinases may be responsible for the observed variations in protein phosphorylation, including PKCs, MAPK, and PKA. This research provides the foundation of breeding techniques for the rapid dissemination of key genes to improve livestock quality and clarifies the usefulness of proteomic methods in diagnosing reproductive potential in the livestock industry. As far as we know, this is the first research that shows a parallel quantitative proteomics and phosphoproteomics-based study of porcine sperm global proteins in different breeds, and these data may supply significant information for understanding the molecular mechanisms underlying the differences in reproductive efficiency among different varieties.

## Data Availability Statement

The datasets presented in this study can be found in online repositories. The names of the repositories and accession numbers can be found below: ProteomeXchange, http://www.proteomexchange.org/, PXD025607 and iProX, https://www.iprox.org/, IPX0003000002.

## Ethics Statement

The animal study was reviewed and approved by Animal Care Commission of the College of Life Science, Xinyang Normal University, China.

## Author Contributions

YX and HX designed the study. YX, QH, and CM performed the research. YW and PZ performed the proteomic technology. YX, PZ, CL, and XC analyzed the data. YX, QH, and HX interpreted the data. YX, QH, CM, and HX drafted the manuscript. All authors critically reviewed and approved the final version of the manuscript.

## Conflict of Interest

The authors declare that the research was conducted in the absence of any commercial or financial relationships that could be construed as a potential conflict of interest.

## Footnotes

1 http://proteomecentral.proteomexchange.org

2 http://www.geneontology.org

3 http://www.kegg.jp

4 http://string-db.org

5 http://www.pantherdb.org/

6 http://www.hprd.org

## Supplementary Material

Supplementary Figure 1 Map of AKAP4 phosphorylation sites identified from porcine boar Spermatozoa. The down-regulated phosphorylated sites are represented in green while the up-regulated ones are colorized in red. Black box represents the phosphorylation motif.

Supplementary Figure 2 GO enrichment analysis of the total identified porcine boar sperm proteins. **(A)** GO cellular compartments categories; **(B)** GO biological process categories; **(C)** GO molecular functions.

Supplementary Figure 3 Western blot result of the selected proteins.
