# Supplementary figures and images for: Comparative Proteomics and Phosphoproteomics Analysis Reveal the Possible Breed Difference in Yorkshire and Duroc Boar Spermatozoa
Source: Front Cell Dev Biol. 2021 Jul 16;9:652809. doi: 10.3389/fcell.2021.652809 (PMC8322956; doi:10.3389/fcell.2021.652809)

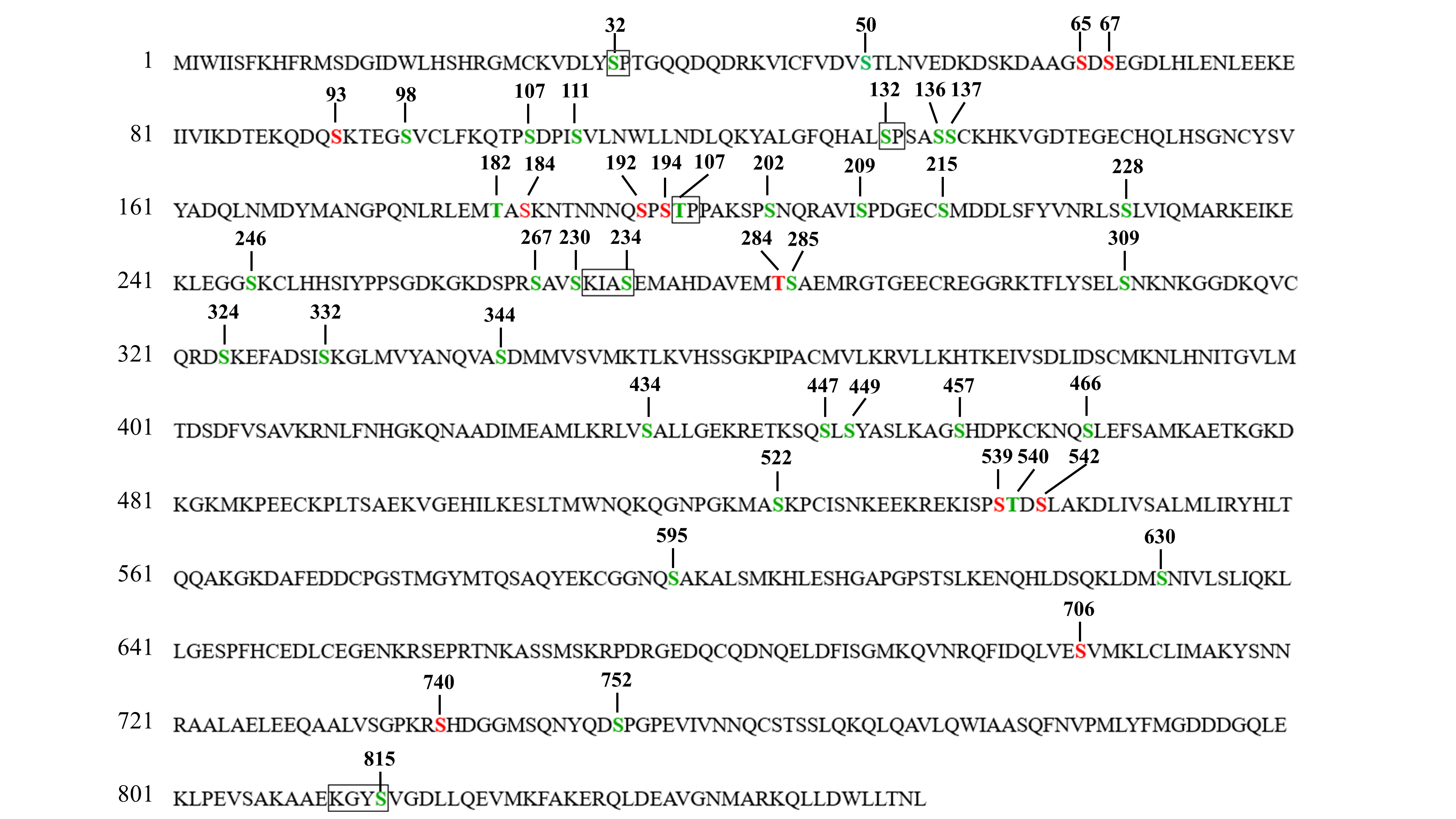

Supplement: Supplementary Figure 1 — Map of AKAP4 phosphorylation sites identified from porcine boar Spermatozoa. The down-regulated phosphorylated sites are represented in green while the up-regulated ones are colorized in red. Black box represents the phosphorylation motif. [file Image_1.TIF]

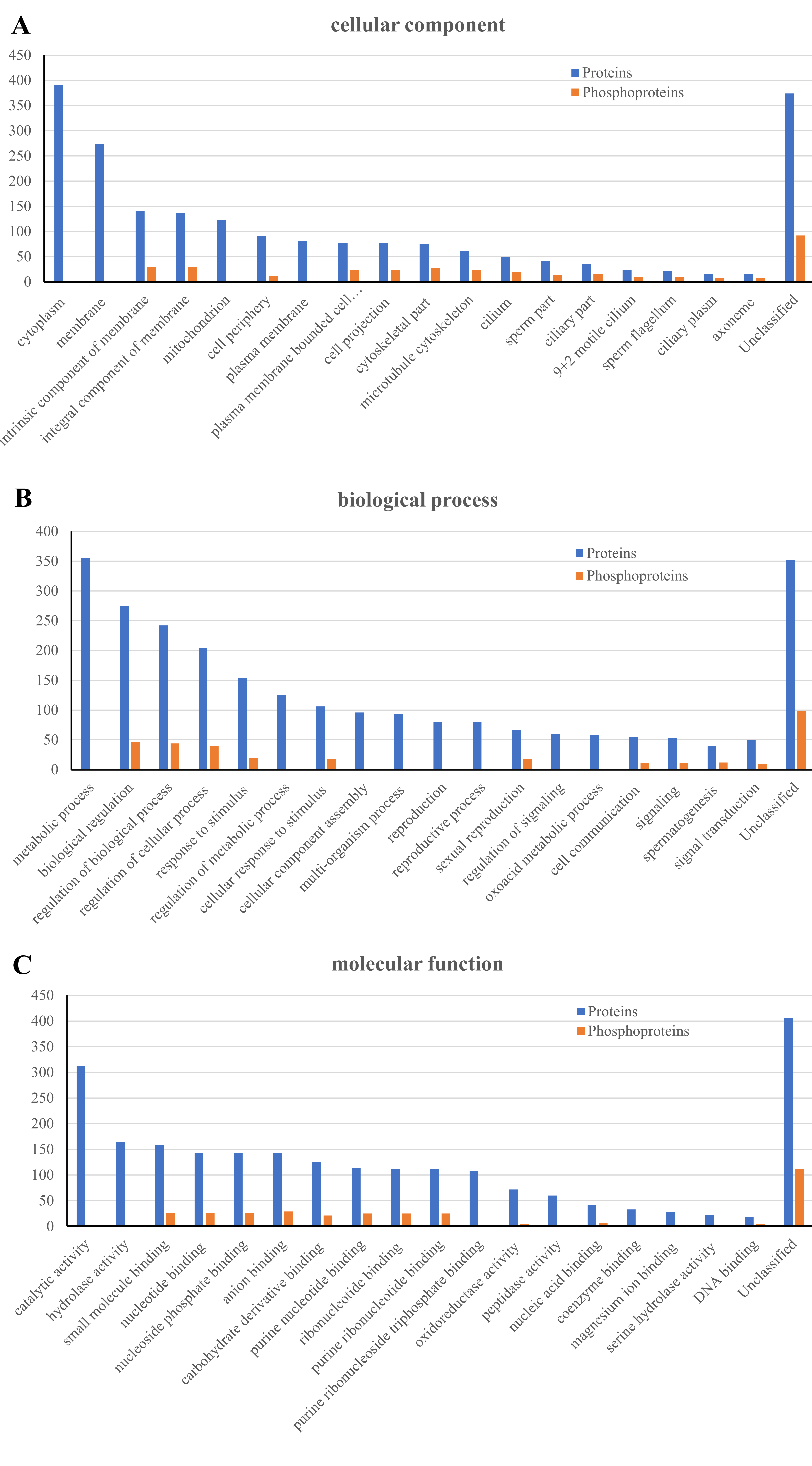

Supplement: Supplementary Figure 2 — GO enrichment analysis of the total identified porcine boar sperm proteins. (A) GO cellular compartments categories; (B) GO biological process categories; (C) GO molecular functions. [file Image_2.TIF]

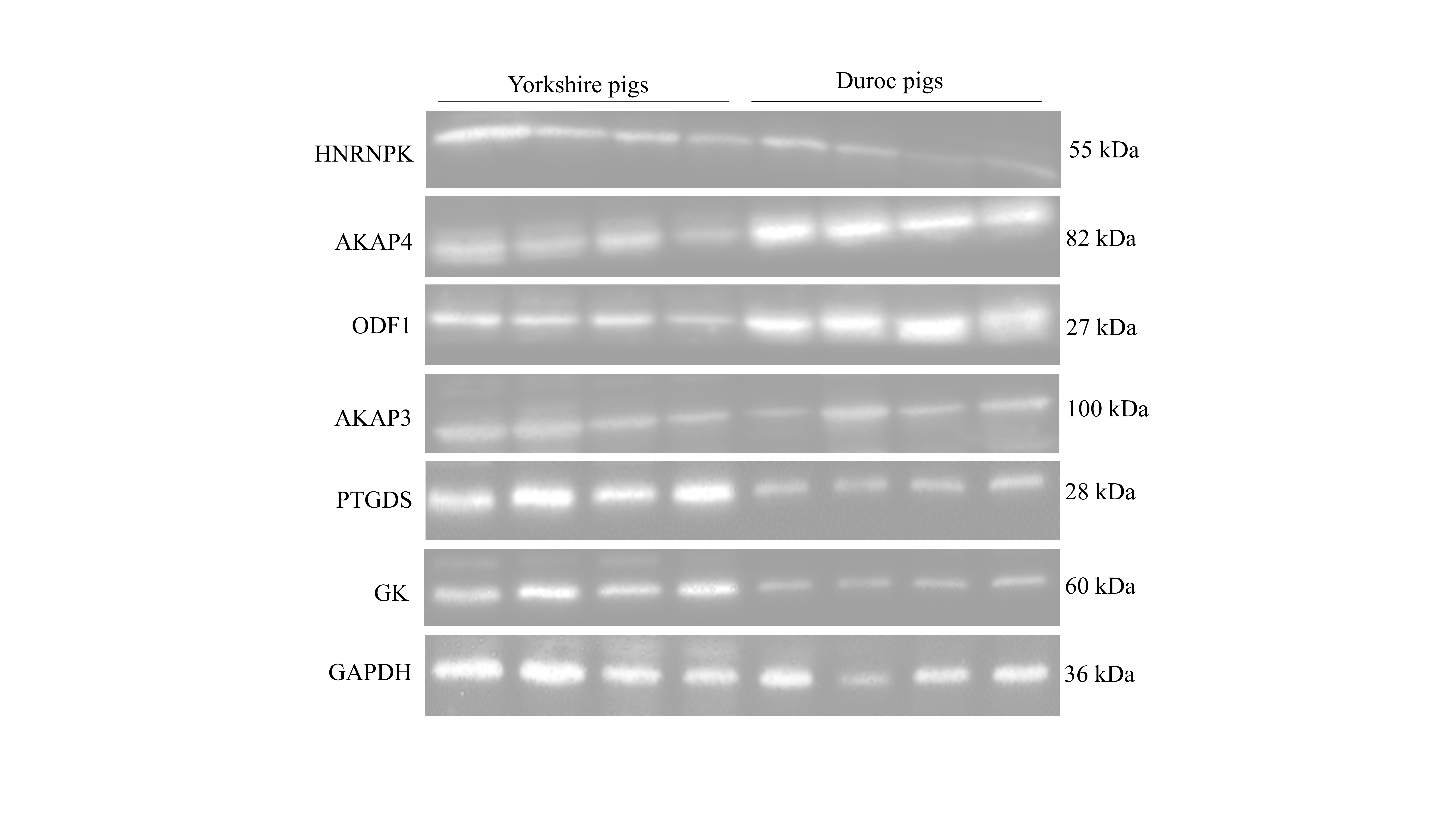

Supplement: Supplementary Figure 3 — Western blot result of the selected proteins. [file Image_3.TIF]
